# Influenza infection elicits an expansion of gut population of endogenous *Bifidobacterium animalis* which protects mice against infection

**DOI:** 10.1186/s13059-020-02007-1

**Published:** 2020-04-28

**Authors:** Qiang Zhang, Jin Hu, Jia-Wu Feng, Xiao-Tong Hu, Ting Wang, Wen-Xiao Gong, Kun Huang, Yi-Xiong Guo, Zhong Zou, Xian Lin, Run Zhou, Yu-Qi Yuan, An-Ding Zhang, Hong Wei, Gang Cao, Chen Liu, Ling-Ling Chen, Mei-Lin Jin

**Affiliations:** 1 National Key Laboratory of Agricultural Microbiology, Huazhong Agricultural University, Wuhan, 430070 People’s Republic of China; 2 Hubei Key Laboratory of Agricultural Bioinformatics, College of Informatics, Huazhong Agricultural University, Wuhan, 430070 People’s Republic of China; 3 Novogene Bioinformatics Institute, Beijing, 100000 People’s Republic of China; 4 College of Veterinary Medicine, Huazhong Agricultural University, Wuhan, 430070 People’s Republic of China; 5 Key Laboratory of Development of Veterinary Diagnostic Products, Ministry of Agriculture, Wuhan, 430070 People’s Republic of China

**Keywords:** Influenza, Gut microbiome, Heterogeneous response, LD_50_, *Bifidobacterium animalis*, Anti-influenza effect, Prognosis predictor

## Abstract

**Background:**

Influenza is a severe respiratory illness that continually threatens global health. It has been widely known that gut microbiota modulates the host response to protect against influenza infection, but mechanistic details remain largely unknown. Here, we took advantage of the phenomenon of lethal dose 50 (LD_50_) and metagenomic sequencing analysis to identify specific anti-influenza gut microbes and analyze the underlying mechanism.

**Results:**

Transferring fecal microbes from mice that survive virulent influenza H7N9 infection into antibiotic-treated mice confers resistance to infection. Some gut microbes exhibit differential features to lethal influenza infection depending on the infection outcome. *Bifidobacterium pseudolongum* and *Bifidobacterium animalis* levels are significantly elevated in surviving mice when compared to dead or mock-infected mice. Oral administration of *B. animalis* alone or the combination of both significantly reduces the severity of H7N9 infection in both antibiotic-treated and germ-free mice. Functional metagenomic analysis suggests that *B. animalis* mediates the anti-influenza effect via several specific metabolic molecules. In vivo tests confirm valine and coenzyme A produce an anti-influenza effect.

**Conclusions:**

These findings show that the severity of influenza infection is closely related to the heterogeneous responses of the gut microbiota. We demonstrate the anti-influenza effect of *B. animalis*, and also find that the gut population of endogenous *B. animalis* can expand to enhance host influenza resistance when lethal influenza infection occurs, representing a novel interaction between host and gut microbiota. Further, our data suggest the potential utility of *Bifidobacterium* in the prevention and as a prognostic predictor of influenza.

## Background

The gut microbiota is a complex community of gut-dwelling bacteria that are central to human health and disease [1, 2]. The maintenance of a mutualistic relationship with the gut microbiota is critical for human health. Disrupting the relationship promotes and even directly causes a variety of diseases and dysfunctions, including inflammatory disorders, colon cancer, and autoimmunity [3]. Although a variety of mechanisms through which gut microbiota protects the host against intestinal infection have been described [4], the mechanisms through which the gut microbiota protects against extraintestinal infection, particularly in the respiratory tract, have not been fully elucidated [5].

Influenza is an acute communicable respiratory illness that affects the upper and lower respiratory passages and severely threatens human health [6]. Annually, influenza epidemics affect close to 1 billion individuals, with 3–5 million severe cases and up to 500,000 fatalities [7]. An example of an influenza virus is H7N9. It occasionally (since 2013) causes epidemic outbreaks in mainland China, Hong Kong, Macau, and Taiwan [8]. The gut microbiota play an important role in protecting the host against influenza virus infection [9–11]. The protective effects of the gut microbiota against influenza have been increasingly investigated in recent years, and several novel mechanisms have been confirmed. For example, commensal microbiota regulates the immune response against influenza by activating the inflammasome via Toll-like receptors [9]. Further, microbial metabolites, such as desaminotyrosine, exert a protective effect against influenza via interferon I signaling [10]. However, despite all of this, the mechanistic details about the anti-influenza effects of gut microbiota remain largely unknown.

Host susceptibility to disease is frequently heterogeneous [10] that is increasingly attributed to the gut microbiota [12–14]. The phenomenon of lethal dose 50 (LD_50_) is a common example of the heterogeneity in the host response to infection, which describes the microbe dose that kills 50% of a host population [15]. Here, to study how gut microbiota affects the host susceptibility to influenza infection, we have used a reverse screening for functional bacteria based on different infection states in mouse models (e.g., ill or healthy survivor of infection, or death upon infection). In particular, based on the LD_50_ concept and metagenomic sequencing analysis, we established murine infection models might be an effective strategy to identify the microbes responsible for the beneficial effect of gut microbiota against influenza infection and the underlying mechanism. Using this approach, we identified variations in the gut microbiota composition and metabolism in mice that responded differently to influenza infection. Furthermore, we found that the transfer of fecal microbes from mice that survived H7N9 virulent infection to antibiotic-treated mice conferred resistance to influenza infection. Finally, we successfully identified *Bifidobacterium animalis* as an anti-influenza species and analyzed the underlying mechanism. Our study not only provides an effective strategy to screen for specific anti-influenza bacteria from microbiota, but also enriched our understanding about the effect of gut microbiota in protecting the host against influenza infection, thus setting the stage for the development of anti-influenza probiotics.

## Results

### Clinical signs and pulmonary histopathological lesions are different in mice that succumbed to or survived the influenza infection

**Fig. 1:**
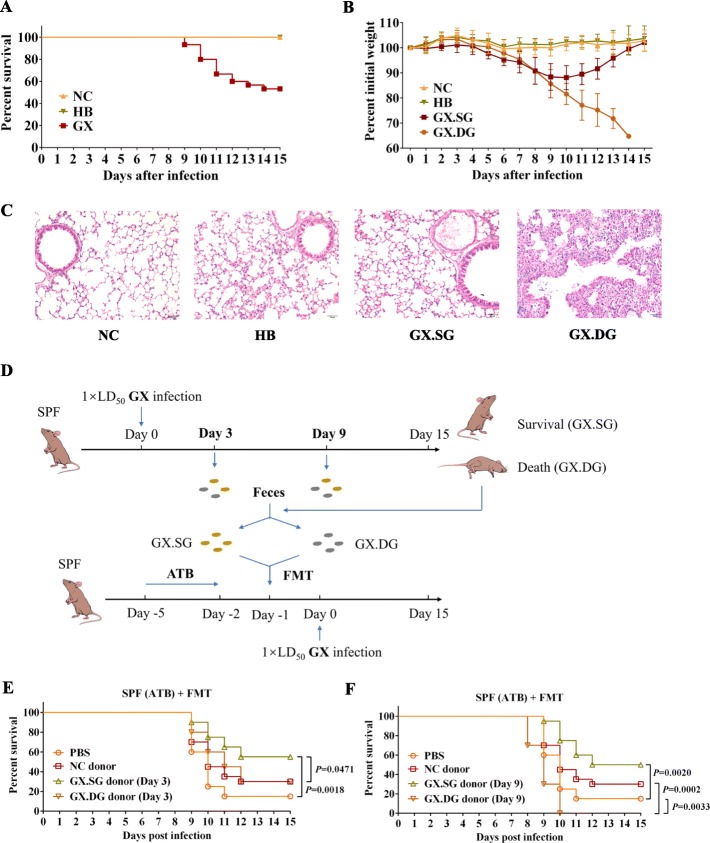
The heterogeneity of mouse response to influenza infection is closely associated with the gut microbiota. **a**, **b** SPF C57BL/6 mice were intranasally infected with a LD_50_ dose of the virulent H7N9 virus strain GX (*n* = 30, 50 μL/mouse), the same dose of the attenuated H7N9 virus strain HB (*n* = 10, 50 μL/mouse), or were administered an equal volume of PBS (NC group) (*n* = 10, 50 μL/mouse). **a** Mouse survival rate. **b** Mouse weight loss. Based on survivability, the GX-infected mice were divided into a group that survived the infection (GX.SG) and a group that succumbed to the infection (GX.DG). The data are presented as the mean ± SD. **c** H&E staining of the mouse lung. The mice were sacrificed on day 10 post-infection of another repeat experiment, and the lungs were collected. The mice in the GX group that succumbed to the infection comprised the GX.DG group (*n* = 3), and the mice in the GX group that started to show improvement were defined as the GX.SG group (*n* = 3), as well as the HB group (*n* = 3) and NC group (*n* = 3). Representative images are shown. **d** Experimental setup for FMT experiments. Fecal microbes were collected on days 3 and 9 after GX infection (*n* = 20) and were divided into the GX.SG (*n* = 10) and GX.DG (*n* = 10) groups based on whether the mice from which the samples were collected succumbed to or survived the infection. SPF mice that had been treated with an antibiotic solution (ATB) for 3 days underwent FMT of the fecal microbes, as indicated. One day after FMT, the mice were infected with a LD_50_ dose of GX. **e**, **f** The survival of GX infection by mice after FMT of feces from GX-infected mice obtained on days 3 (**e**) or 9 (**f**) after the infection or feces from NC mice; survival of mice administered PBS before GX infection is shown as a control (*n* = 20 mice per group). Statistics in survival assay were done with the log-rank (Mantel-Cox). All experiments were performed at least twice under similar conditions and yielded similar results

To assess the heterogeneity in the host response to influenza, we infected specific pathogen-free (SPF) C57BL/6 mice with virulent (GX) and attenuated (HB) H7N9 virus using the LD_50_ approach (see the “Materials and methods” section for the two virus details). Consequently, C57BL/6 mice were infected with LD_50_ dose of the virulent H7N9 strain (the GX group), the same load of attenuated virus (the HB group), or administered phosphate-buffered saline (PBS) (the negative control NC group). Animal body weights and survival were monitored, and fecal samples were collected daily after the infection. All mice in the GX group exhibited obvious clinical signs of infection, including weight loss, anorexia, shivering, and a rough hair coat. The survival rate in this group was 53.3% (16/30) (Fig. 1a). By contrast, all mice in the NC and HB groups survived and exhibited no obvious symptoms of infection. Further, based on whether they succumbed to or survived from the infection, the mice in the GX group were divided into the death group (*n* = 14, GX.DG) and survival group (*n* = 16, GX.SG), respectively. Of the GX.SG group, 10 mice were randomly selected from the 16 mice as the representative of this group. The percentile curves for the mouse body weights revealed that the mice in the GX.DG group lost more body weight than those in the GX.SG group (Fig. 1b). To further evaluate the differences between the GX.DG and GX.SG groups, a repeated experiment was performed. At day 10 post-infection, the mice in the GX group that died formed the GX.DG group, whereas those mice that survived and started to show signs of improvement comprised the GX.SG group. Indeed, both autopsy and histological examination revealed that the lung lesions in the GX.DG mice were notably more severe than those in the GX.SG mice (Fig. 1c and Additional file 1: Fig S1). Taken together, these findings demonstrated that mice that succumbed to or survived infection with the same dose of influenza virus have clearly different clinical signs and obvious histopathological differences, representing a heterogeneous response to infection and confirming that the LD_50_ approach can be used to differentiate between individual-specific responses to infection.

### The transfer of fecal microbiota from mice that survived virulent influenza infection increases the resistance of the recipient mice to influenza

To investigate whether the differences in the responses of mice to influenza infection are associated with the gut microbiota, we next performed fecal microbiota transplantation (FMT) (Fig. 1d). The effect of fecal microbiota transferred from mice that succumbed to or survived the virulent infection on infection with the GX virus in recipient mice was then assessed. FMT of the fecal microbiota collected from GX.SG mice on either day 3 or 9 post-infection significantly increased the survival of the recipient mice to influenza infection compared with the resistance of mice transplanted with fecal microbiota from uninfected mice (NC donor) or that of PBS-treated mice (Fig. 1e, f). By contrast, FMT of the fecal microbiota collected on day 9 post-infection from GX.DG mice reduced the survival rate of the recipient mice after influenza infection (Fig. 1f). These findings indicated that the fecal microbiota of mice that survived the infection likely contains specific intestinal microbes that provide protection against influenza.

### Gut microbiota exhibits obvious differential characteristics depending on the severity of influenza infection

**Fig. 2:**
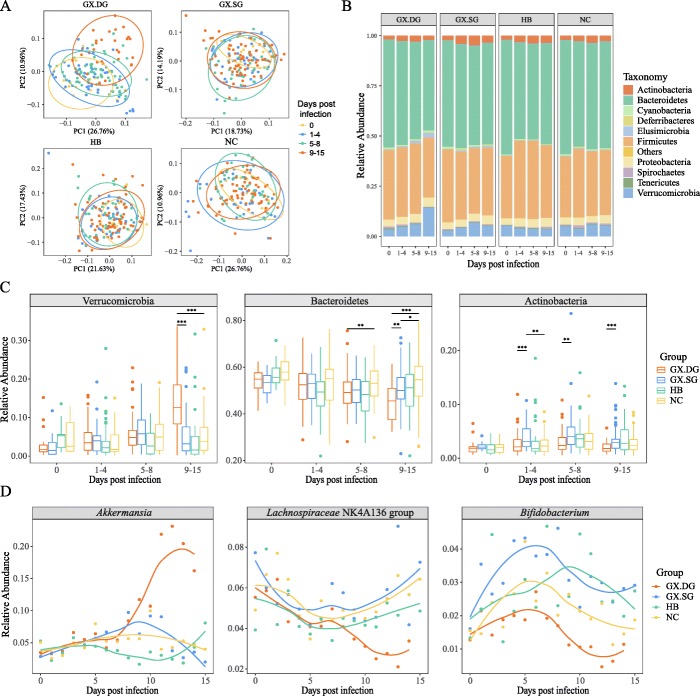
The influenza virus infection alters the gut microbiota to various extent in individual mice. The fecal samples were collected on days 0–15 post-infection in the experiment shown in Fig. 1a, b. Based on survivability, the GX-infected mice were divided into a group that survived the infection (GX.SG) and a group that succumbed to the infection (GX.DG). All fecal samples were also divided into the corresponding groups. The GX.DG, GX.SG, NC, and HB groups contained 156, 160, 160, and 160 fecal samples for 16S rRNA gene sequencing analysis, respectively. **a** PCoA of the weighted UniFrac distances among the GX.DG, GX.SG, HB, and NC groups. **b** Relative abundances of bacterial phyla in the GX.DG, GX.SG, HB, and NC groups. **c** Bacterial phyla showing significant differences in abundance (*P < 0.05, **P < 0.01, ***P < 0.001, Wilcoxon-Mann-Whitney test). To more intuitively analyze the data (**a**–**c**), samples from the 16 time points were grouped into four infection stages: stage 1 (day 0), control; stage 2 (days 1–4), the stable weight stage; stage 3 (days 5–8), the weight loss stage; and stage 4 (days 9–15), the death stage. **d** Trends of the abundance changes of several bacteria at the genus level

To identify the anti-influenza intestinal microbes of mice that survived the infection, we next used 16S rRNA gene sequencing to analyze the fecal microbiota of mice daily, during the 16 days that the experiment lasted. To more intuitively analyze the data, samples from the 16 time points were grouped into four infection stages: stage 1 (day 0), control; stage 2 (days 1–4), the stable weight stage; stage 3 (days 5–8), the weight loss stage; and stage 4 (days 9–15), the death stage. The Shannon diversity index analysis indicated that the abundance and diversity of the bacterial community followed a decreasing trend at stage 4 in the GX.DG mice but not in the GX.SG, HB, or NC mice (Additional file 2: Fig S2). Further, the principal coordinate analyses (PCoAs) of weighted UniFrac distances indicated a notable shift in the microbiome community structure in the GX.DG samples with disease progression, particularly at stage 4, whereas only a slight shift was observed in the GX.SG and HB groups. By contrast, almost no shift was observed in the NC group (Fig. 2a). An intergroup analysis showed similar results: the sample clustering at the last infection stage revealed differences between the GX.DG group and the other three groups (Additional file 3: Fig S3).

Furthermore, a detailed phylum-level taxonomic analysis of the intestinal microbiota revealed obvious changes in the bacterial taxonomic composition in the gut microbiota of mice infected with the GX strain, particularly in the GX.DG mice (Fig. 2b). Several phyla exhibited similar variation trends in the GX.DG and GX.SG groups; for example, in both groups of mice, *Elusimicrobia* and *Proteobacteria* became more abundant than in the NC group, while *Bacteroidetes* became less abundant than in the NC group as infection proceeded, although the changes in the *Elusimicrobia* abundance were more notable (Fig. 2c and Additional file 4: Fig S4). Aside from these similarities, several differences were apparent between the GX.DG and GX.SG groups: for example, during the infection, *Verrucomicrobia* abundance significantly increased only in the GX.DG group, while *Actinobacteria* abundance clearly increased only in the GX.SG group, compared with the NC group (Fig. 2c). In addition, the variation trends of *Firmicutes* abundance visibly fluctuated, with higher abundance in GX.DG than in GX.SG at the third stage post-infection, but the other way around at the fourth stage (Additional file 4: Fig S4). These results indicated that infection with the virulent H7N9 virus strain significantly changed the composition of the gut microbiota, with clear differences between infected vs. non-infected as well as dead vs. survived mice groups.

To further detail the effect of influenza infection on the gut microbiota, we performed taxonomic analyses of the intestinal microbiota at the genus level. Based on the variation trends of gut microbes in the four mouse groups, the differentially abundant microbes were divided into six groups (Fig. 2d and Additional file 5: Fig S5): (1) bacteria with increased abundance only in the GX.DG group (*Akkermansia*, *Bacteroides*, *Parabacteroides*, unidentified *Gastranaerophilales*, *Butyricimonas*, and unidentified *Ruminococcaceae*), (2) bacteria whose abundance first increased and then decreased only in the GX.DG group (*Enterorhabdus*, *Adlercreutzia*, and *Candidatus Saccharimonas*), (3) bacteria with increased abundance in both the GX.DG and GX.SG groups (*Elusimicrobium*, *[Eubacterium]_coprostanoligenes_group*, and *Ruminococcaceae_UCG-005*), (4) bacteria whose abundance decreased only in the GX.DG group (*Lachnospiraceae_NK4A136_group*, *Lactobacillus*, and *Parasutterella*), (5) bacteria whose abundance increased in the GX.DG and GX.SG groups and particularly in the HB group (*Turicibacter* and *Allobaculum*), and (6) bacteria whose abundance increased in the GX.SG group and decreased in the GX.DG group (*Bifidobacterium*). The analysis indicated different genera of the gut microbiota exhibit differential responses to influenza infection, with some common features shared by individual genus depending on the outcome of the infection. Based on the correlations between the abundances of gut microbes and the health status of the infected mice, we speculated that *Lachnospiraceae_NK4A136_group*, *Parasutterella*, *Lactobacillus*, and *Bifidobacterium* might play important roles in the host’s defense against influenza infection.

### *Bifidobacterium pseudolongum* and *Bifidobacterium animalis* are strongly associated with the survivability of influenza-infected mice

**Fig. 3:**
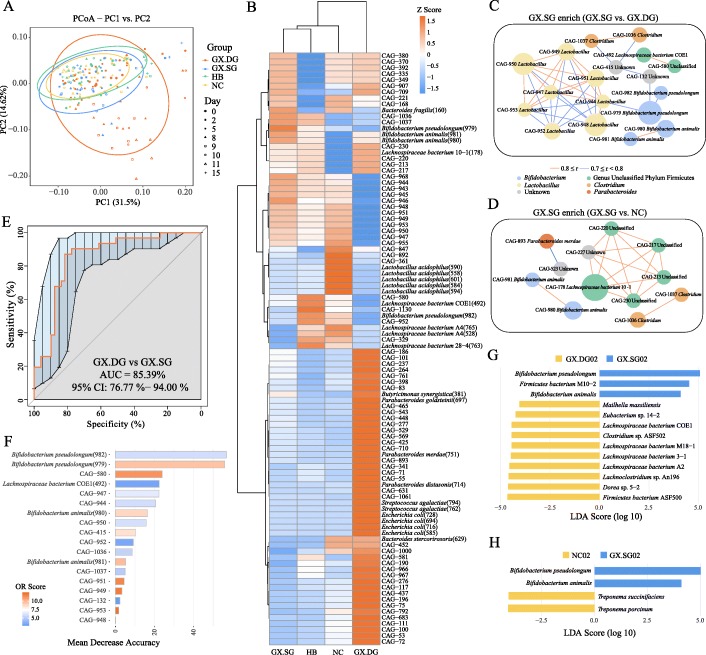
The survivability of infected mice is closely associated with the enrichment of several bacterial species. Experimental description references to Fig. 2. Of which, 177 representative samples from all groups collected on days 0, 2, 5, 8, 9, 10, 11, and 15 post-infection (57 samples from the GX.DG group and 40 samples each from the GX.SG, NC, and HB groups) were selected for metagenomic sequencing analyses. **a** PCoA of the weighted UniFrac distances among the GX.DG, GX.SG, HB, and NC groups. **b** Heat map of the relative abundances of CAGs. **c**, **d** Co-occurrence networks generated from the comparisons of the GX.SG group with the GX.DG (**c**) and NC (**d**) groups. The co-occurrence network inferred for each fecal sample according to Spearman’s correlations was stratified by the infection type. Each node in the network indicates a CAG, and the node size represents the average relative abundance of one CAG. Only the CAGs enriched in the GX.SG group compared with the GX.DG or NC group (OR > 2) were retained. **e** ROC curve of the final random forest model constructed using the relative abundances of 18 CAGs enriched in the GX.SG group compared with the GX.DG group. The AUC statistic is a summary measure of classifier performance. AUC values close to 1 indicate that a high true positive rate was achieved with a low false-positive rate (ideal performance), whereas AUC values close to 0.5 indicate random performance. **f** Variable importance according to the mean decrease in accuracy of the random forest classifier. The mean decrease in accuracy obtained after the permutation of a variable shows the importance of the variable with respect to its contribution to the accuracy of the random forest classifier. Variables with a high mean decrease in accuracy contribute more to the classification of the data than variables with a low mean decrease in accuracy. **g**, **h** LEfSe comparison of gut microbes at the species level between the GX.SG and GX.DG groups (**g**) and the GX.SG and NC groups (**h**) on day 2 post-infection

Since the above taxonomic analysis indicated that specific bacterial genera could impact the host’s response to infection, we moved on to analyze the bacteria at the species level. Accordingly, 177 representative samples from all groups collected on days 0, 2, 5, 8, 9, 10, 11, and 15 post-infection were selected for metagenomic sequencing analyses. For gene richness characterization, a rarefaction analysis was performed. The estimated gene richness values almost approached saturation in all groups, indicating that the sequencing data had sufficient coverage and that only very few genes may be undetected (Additional file 6: Fig S6). The results of PCoA analysis showed a notable shift in the gut microbiota composition in the GX.DG samples at the later infection stage (Fig. 3a), consistent with those of the 16S rRNA gene sequencing analysis. This indicated that the starting dataset could be used for meaningful analyses of changes in bacterial abundance at the species level.

We then analyzed the co-abundance gene groups (CAGs) of all genes to uncover any correlations between species abundance and the host’s health status. Overall, 1157 CAGs were identified, and 98 of these groups were annotated (Additional file 7: Table S1). The relative abundance of the annotated CAGs in the four mouse groups is shown in Fig. 3b. Indeed, CAGs enriched in the GX.DG group differed from those in the NC group: the former exhibited greater abundance of *Escherichia coli*, *Bacteroides*, *Parabacteroides*, *Streptococcus agalactiae*, and *Helicobacter* and lower abundance of *Lactobacillus* (CAGs of these bacteria were listed in Additional file 7: Table S1). Although the CAG components in the GX.SG and HB groups were very similar to those in the NC group, several specific components were identified. For example, the GX.SG group exhibited greater abundances of *B. pseudolongum* and *B. animalis*, and the HB group was enriched in *Lachnospiraceae*. For the comparative analysis of the GX.SG and GX.DG groups, or the GX.SG and NC groups, the GX.SG-associated CAGs were classified as enriched in the GX.SG group (odds ratio (OR) score > 2). To further identify the dominant specific species in the GX.SG group and their correlations with disease severity, Spearman’s correlation network of the CAGs enriched in the GX.SG group was generated (data for days 9–15 are shown in Fig. 3c, d). Compared with the GX.DG group, the GX.SG group exhibited a greater abundance of 18 CAGs (Fig. 3c), most of which were previously found to confer health benefits [16, 17]. In addition, compared with the NC group, the GX.SG group was enriched in 12 CAGs (Fig. 3d), of which *B. animalis* (2 CAGs) and *Clostridium* sp. (2 CAGs) were shared with the above correlation network. This indicated these two species might play an important role (e.g., protective role) during influenza infection. Next, a random forest classifier was used to classify the GX.SG and GX.DG groups using the 18 CAGs enriched in the GX.SG group compared with the GX.DG group. Tenfold cross-validation was repeated five times, and the area under the curve (AUC) of receiver operating characteristic (ROC) was used as the scoring method to evaluate the accuracy of the classifier on a testing dataset (Fig. 3e). Based on the test set, an average AUC of 85.39% was obtained with a 95% confidence interval (CI) of 76.77 to 94%, indicating that this model has a powerful diagnostic potential for predicting infected mice’s prognosis/severity. Moreover, the contribution of each CAG was evaluated based on the mean decrease in accuracy (Fig. 3f). Among the 18 CAGs analyzed, the CAGs annotated as *B. pseudolongum* (CAG-982 and CAG-979), *Lactobacillus* sp. (CAG947, CAG944, CAG950, and CAG952), and *B. animalis* (CAG980 and CAG981) contributed most to the identification of the GX.SG and GX.DG groups. These findings further confirmed the close correlation between the survivability of infected mice and the presence of specific bacteria, especially *B. pseudolongum*, *Lactobacillus*, and *B. animalis*.

Further, to get the detailed differences of the gut microbiota at each time point, the effect size analysis (LEfSe) of linear discriminant analysis (LDA) was performed (Fig. 3g, h and Additional file 8: Fig S7). At day 0 post-infection, the abundances of gut microbes have no apparent differences between the groups GX.SG and GX.DG or NC. However, the abundances of *B. pseudolongum* and *B. animalis* were obviously increased in the GX.SG mice at 2 days post-infection compared with their abundances in the GX.DG or NC mice (Fig. 3g, h). The elevated distribution of *B. pseudolongum* in GX.SG mice persisted throughout the infection process, with *B. animalis* also found at elevated levels at days 10 and 11 post-infection (Additional file 8: Fig S7). In addition, to validate the metagenomic analysis, qRT-PCR was performed to detect *B. pseudolongum* and *B. animalis* of fecal samples collected from another repeated experiment. As shown in Additional file 9: Fig S8, the populations of *B. pseudolongum* and *B. animalis* were actually enriched in GX.SG vs. in GX.DG at day 2 and day 10 post-infection, but not at day 0 post-infection. These suggested that the variation in the abundances of *B. pseudolongum* and *B. animalis* between the GX.DG and GX.SG groups was associated with the differential responses of these mice to influenza infection and not due to the differences in the initial abundances of these gut microbes.

Based on the objective cause-precedes-effect law in a causal relationship, and on the above-described FMT experiments and distribution characteristics of gut microbes in the GX.SG group, we hypothesized that the gut microbiota increases the resistance of the host mouse to virulent H7N9 virus infection by increasing the abundance of endogenous *B. pseudolongum* and/or *B. animalis*.

### Gavage of survival-associated microbes protects the host against influenza

**Fig. 4:**
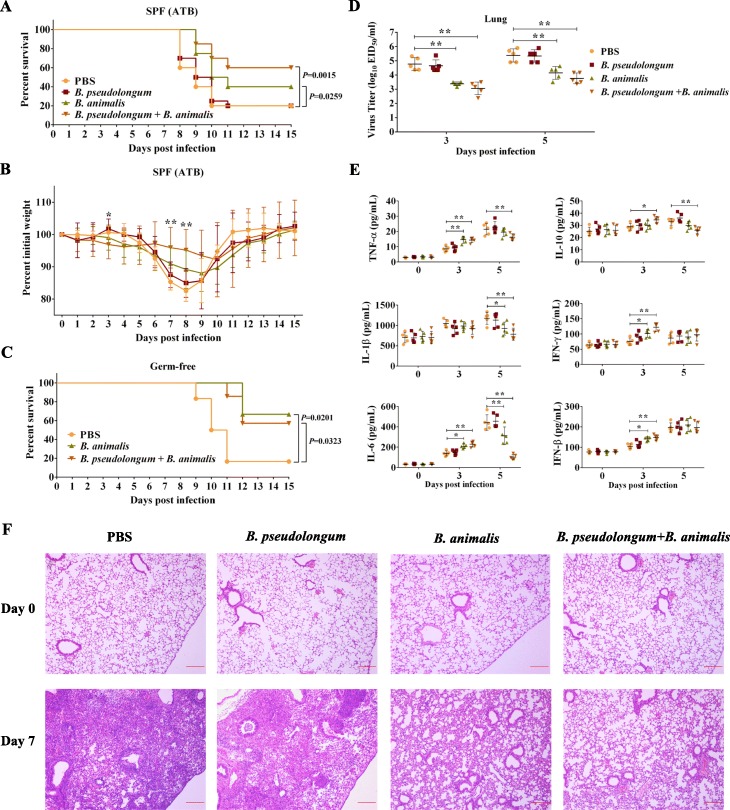
The oral administration of survival-associated microbes protects the recipient against virulent H7N9 virus infection. Survival rate (**a**) and weight loss (**b**) of mice treated with an antibiotic solution (ATB), administered survival-associated microbes or PBS, and then infected with GX (*n* = 20 mice per group). The administered microbes were *B. pseudolongum* alone, *B. animalis* alone, or a combination of *B. pseudolongum* and *B. animalis*. Significant difference of weight changes (*P* < 0.05) only occurred between group combination gavage and PBS gavage on days 3, 7, and 8. **c** Survival rate of germ-free mice administered *B. animalis* alone (*n* = 6), the two-bacterium combination (*n* = 7), or PBS (*n* = 6) and then infected GX. Virus titers (**d**), cytokine concentrations (**e**), and histological examination (H&E staining) (**f**) of the lungs of ATB-pretreated mice after GX infection. Before infection, the ATB-pretreated mice were administered with *B. pseudolongum* alone, *B. animalis* alone, the two-bacterium combination, or PBS, with 15 mice in each group. Five mice per group were randomly sacrificed for the collection of the lungs on days 0, 3, and 5 post-infection. The lung homogenate was prepared for the determinations of virus titers and cytokine concentrations. In addition, 24 ATB-pretreated mice were randomly divided into four groups and also treated as described above. On days 0 and 7 post-infection, 3 mice per group were randomly sacrificed. The lungs were collected for the histological examination. Representative images are shown. The data are presented as the mean ± SD. Statistics for weight changes, virus titers, and cytokine concentrations were two-way ANOVA. *P < 0.05, **P < 0.01. Statistics in survival assay were done with the log-rank (Mantel-Cox). All experiments were performed at least twice under similar conditions and yielded similar results

To verify the role of *B. pseudolongum* and *B. animalis* on host resistance to influenza infection, SPF mice were treated with an antibiotic solution (ATB) [18] and then administered the two bacterial strains (in single or in combination) by oral gavage before infection. Oral administration of *B. animalis* alone or two-bacterium combination significantly increased the survival rate of recipient mice, but gavage with *B. pseudolongum* alone had no obvious effect (Fig. 4a). Further, the weight loss was most clearly improved on days 6–10 after the infection by gavage with the two-bacterium combination, and somewhat improved by gavage with *B. animalis* alone (Fig. 4b). To eliminate any possible artifacts (e.g., residual microflora) associated with the ATB-treated mice, germ-free mice were orally administered with *B. animalis* alone, two-bacterium combination, or PBS. Similarly, gavage with *B. animalis* alone and two-bacterium combination both significantly increased the survival of germ-free mice. The only difference is that in germ-free mice, the administration with *B. animalis* alone provided almost equal protection as the combination gavage, but in ATB-pretreated mice, there was only half as much protection by gavage with *B. animalis* compared to combination gavage (Fig. 4c). These observations demonstrated that the transfer of *B. animalis* alone provided obvious protection against influenza and that this protection could be enhanced by the additional administration of *B. pseudolongum* in non-sterile mice.

A parallel experiment involving ATB-pretreated mice as described above was performed to determine the lung virus titer and cytokine levels and to examine histological changes. On days 3 and 5 post-infection, the lung virus titers in mice administered with *B. animalis* alone or the two-bacterium combination were significantly lower than those in PBS-treated mice (Fig. 4d). However, the cytokine levels in the lungs of mice administered with *B. animalis* alone or the two-bacterium combination showed different trends between the two time points compared to those in mice administered with PBS (Fig. 4e): the levels of the majority of tested cytokines were significantly elevated in the lungs of mice administered with *B. animalis* alone or the two-bacterium combination on day 3 post-infection; by contrast, on day 5 post-infection, the levels of several cytokines in these two groups, especially the two-bacterium combination group, became lower than those in PBS-treated mice. Although only IL-6 levels significantly decreased in the blood of mice administered with *B. animalis* alone and the two-bacterium combination on day 5 post-infection, most cytokines displayed an obvious increase in these two groups on day 3 post-infection (Additional file 10: Fig S9). The data indicated that the lower lung virus titers in mice administered with *B. animalis* alone or the two-bacterium combination compared with those in mice administered with PBS were accompanied by a greater induction of cytokines at the early phase of H7N9 infection. This suggested that *B. animalis* likely protects hosts against influenza infection by modulating the host immune response.

In addition, histological examination revealed that the lungs of mice administered with PBS or *B. pseudolongum* alone displayed severe pathologies on day 7 post-infection, including congestion, inflammatory cell infiltration, and deciduous cells in the bronchial lumen, even the alveoli almost disappeared. By contrast, the lung lesions of mice administered with *B. animalis* alone or the two-bacterium combination were obviously improved (Fig. 4f). This further confirmed the anti-influenza effect of *B. animalis*.

### *B. animalis* contributes specific metabolic functions to the gut microbiome to protect the host against influenza infection

**Fig. 5:**
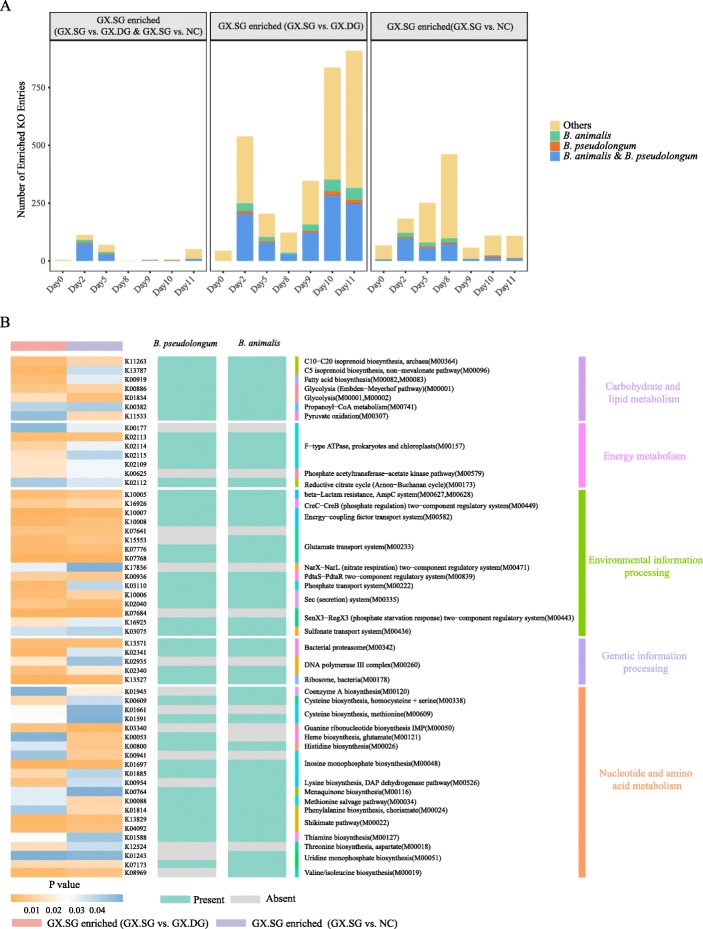
Functional metagenomic comparison of the gut microbiome in different mouse groups reveals that *B. animalis* may mediate a protective effect against influenza by the specific metabolic pathways. **a** Distribution of all KO terms enriched in the GX.SG group compared with the GX.DG or NC group. The yellow color indicates the KO terms related to neither *B. animalis* nor *B. pseudolongum*; the green color indicates the KO terms related to *B. animalis* but not to *B. pseudolongum*; the red color indicates the KO terms related to *B. pseudolongum* but not to *B. animalis*; the blue color indicates the KO terms related to both *B. animalis* and *B. pseudolongum*. **b** Functional composition of the KO terms enriched on day 2 post-infection in the GX.SG group compared with both the GX.DG and NC groups and contributions of *B. animalis* or *B. pseudolongum* to these functions. Only the KO terms labeled with a module (M) number are shown

To further understand the mechanism through which *B. animalis* mediates the anti-influenza effect, we performed a functional metagenome comparison of the gut microbiome between the GX.SG and GX.DG or NC groups. Overall, 2278 KEGG Orthology (KO) terms were enriched in the GX.SG group compared with the GX.DG or NC groups (Additional file 11: Table S2). The distribution characteristics of these KO terms at each time point are shown in Fig. 5a. The comparison of the GX.SG and GX.DG groups revealed an enrichment of KO terms in the GX.SG group mainly at the early and late stages of infection. However, in the comparison of the GX.SG and NC groups, the KO terms enriched in the GX.SG group were mainly observed at the early and middle stages of infection. Further, the number of enriched KO terms in the first comparison (1606) was clearly higher than that in the second comparison (1061). This suggested that the functional structure of the gut microbiota in the GX.SG group was more similar to that in the NC group.

Further, the enriched metabolic pathways in the GX.SG group revealed by the comparisons between the GX.SG and GX.DG groups as well as between the GX.SG and NC groups displayed similar distribution characteristics as those found in the analysis of KO terms (Additional file 12: Fig S10). More importantly, on days 2 and 5 post-infection, many KO terms were enriched in the GX.SG group compared with both the GX.DG and NC groups (double-positive enriched KO terms) (Fig. 5a), implying these KO terms very likely play important roles in defense against influenza. Our above data demonstrated that *B. animalis* was significantly enriched in the GX.SG mice at 2 days post-infection compared with the GX.DG or NC mice, but not at 5 days post-infection (Fig. 3g, h and Additional file 8: Fig S7), suggesting that *B. animalis* provides more anti-influenza effect at 2 days post-infection than at 5 days. Thus, we subsequently analyzed the functional composition of the double-positive enriched KO terms at 2 days post-infection and the contribution of *B. animalis* to these terms/functions. As shown in Fig. 5b, 39 functions were identified among the double-positive enriched KO terms (here, only the KO terms labeled with a module number were shown; other KO terms without module number were listed in Additional file 11: Table S2), which involved five pathways, including carbohydrate and lipid metabolism, energy metabolism, environmental information processing, genetic information processing, and nucleotide and amino acid metabolism. *B. animalis* or *B. pseudolongum* participated in most of these 39 functions and all of the five pathways. Of these pathways, valine/isoleucine biosynthesis (M00019), lysine biosynthesis and the DAP dehydrogenase pathway (M00526), and coenzyme A (CoA) biosynthesis (M00120) are uniquely enriched in *B. animalis*. These results indicated that *B. animalis* may mediate a protective effect against influenza through promoting the biosynthesis of valine, isoleucine, lysine, and CoA.

### Oral administration of valine or intraperitoneal injection of CoA protects the host against influenza

**Fig. 6:**
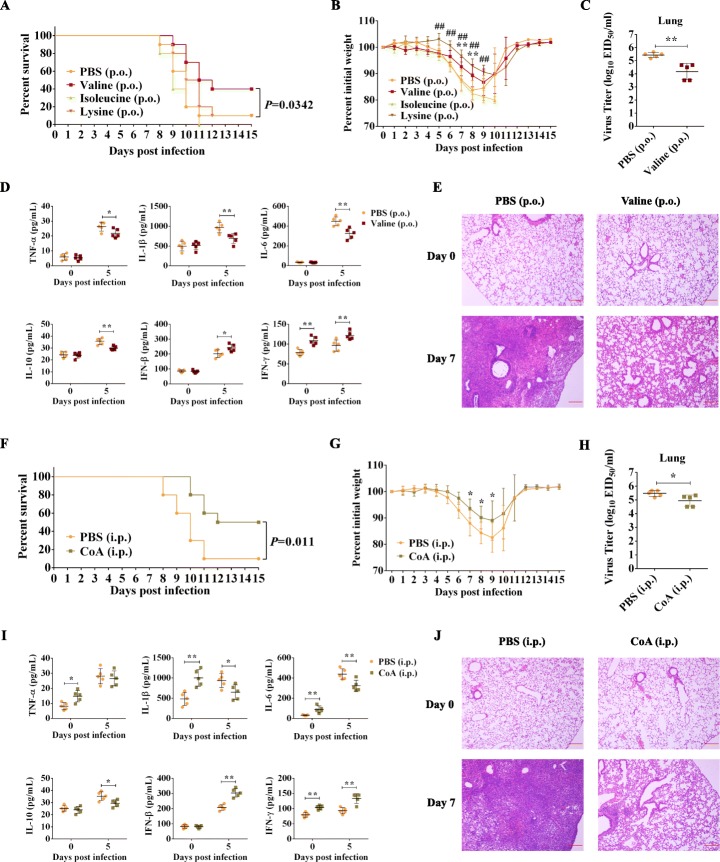
Oral administration of valine or intraperitoneal injection of CoA protect recipient mice against virulent H7N9 virus infection. Survival rate (**a**) and weight loss (**b**) of mice orally administrated (p.o.) daily for 1 week with 100 mg of valine, isoleucine, or lysine, or the same volume of PBS (200 μL) and then infected with H7N9 influenza virus GX (*n* = 10 mice per group). **P < 0.01 (valine vs. PBS), ^**##**^*P* < 0.01 (lysine vs. PBS). Virus titers (**c**), cytokine concentrations (**d**), and histological examination (H&E staining) (**e**) of the lungs of valine-pretreated mice after GX virus infection. *P < 0.05, **P < 0.01 (valine vs. PBS). Survival rate (**f**) and weight loss (**g**) of mice intraperitoneally injected (i.p.) daily for 3 days with 0.2 mg of CoA or the same volume of PBS (100 μL) and then infected with H7N9 influenza virus GX (*n* = 10 mice per group). *P < 0.05 (CoA vs. PBS). Virus titers (**h**), cytokine concentrations (**i**), and histological examination (H&E staining) (**j**) of the lungs of CoA-pretreated mice after GX virus infection. *P < 0.05, **P < 0.01 (CoA vs. PBS). At each time point, 5 mice per group for the detection of virus titers and cytokine concentrations, and 3 mice per group for the histological examination. Statistics in survival assay were done with the log-rank (Mantel-Cox). Statistics for weight changes and cytokine concentrations were two-way ANOVA. Statistics for virus titer was *t* test. The data are presented as the mean ± SD. For the histological examination, representative images are shown. All experiments were performed at least twice under similar conditions and yielded similar results

To verify the above functional metagenomic analysis, ATB-pretreated mice were gavaged with valine, isoleucine, or lysine, or were intraperitoneally injected with CoA, and then intranasally challenged with the H7N9 virus. As shown in Fig. 6a, b, f, g, oral administration of valine or intraperitoneal injection of CoA significantly increased the survival rate of recipient mice and improved the weight loss. By contrast, oral administration of lysine only improved the weight loss, and oral administration of isoleucine had no effect. These results demonstrated that oral administration of valine or intraperitoneal injection of CoA can provide protection in vivo against influenza. However, in vitro, both valine and CoA showed no obvious anti-influenza effect in A549 cells (Additional file 13: Fig S11), suggesting that the anti-influenza effects of valine and CoA might be indirect. To further understand how valine and CoA mediate the anti-influenza effect in vivo, the parallel animal experiments were performed. Then, the lungs were collected to determine virus titer, cytokine levels, and histological changes (Fig. 6c–e, h–j). On day 5 post-infection, both oral administration of valine and intraperitoneal injection of CoA can significantly reduce virus titers and levels of IL-1β, IL-6, and IL-10 of the lungs and at the same time upregulate IFN-β and IFN-γ of the lungs. In addition, on day 7 post-infection, the treatments of both valine and CoA also alleviated the lung lesion compared with that of PBS. The difference is that, on day 0 post-infection, intraperitoneal injection of CoA can obviously induce the expressions of TNF-α, IL-1β, IL-6, and IFN-γ in the lungs, but oral administration of valine only stimulates IFN-γ. These data suggested that the anti-influenza effect of valine could be due to its immunoregulatory properties after influenza infection, by contrast, that of CoA could be because it stimulates the innate immune response in advance.

## Discussion

There is accumulating evidence indicating that the gut microbiota profoundly influences the pathogenicity of influenza virus; however, mechanistic details remain largely unknown [10]. Therefore, the characterization and identification of bacteria that exert anti-influenza effects are warranted, but the lacking of an effective screening strategy to identify specific anti-influenza bacteria from gut microbiota poses a serious challenge. LD_50_ approach can produce a controlled gradient of heath states after disease infections [15], thus making it possible to perform an anti-disease analysis of gut microbes. Here, by combining the LD_50_ approach with metagenomic sequencing analysis, we identified a series of specific characteristics of gut microbes that are closely related to survived and dying fates of infected hosts, e.g., *Bifidobacterium* genus was elevated in survived mice but repressed in dead mice, compared with the mock-infected group (Fig. 2d). However, this close correlation between *Bifidobacterium* genus and the survivability of infected mice disappeared when the individual variability within survived and dead mice was ignored and the data were merged into a single group (GX infection group) (Additional file 14: Fig S12). This indicates that ignoring the individual variability in studies of microbiota leads to the omission of important information and likely explain why the anti-influenza potential of *Bifidobacterium* was not discovered in previous studies of influenza and gut microbiota [19]. Ultimately, taking advantage of the above approach, we successfully identified several potential anti-influenza gut microbes and demonstrated the anti-influenza effect of *B. animalis* in both antibiotic-treated and germ-free mice. These results confirm that our approach is a directional and effective strategy to screen specific anti-influenza bacteria from the gut microbiota. Certainly, LD_50_ is not a unique entry point; any other factor that introduces heterogeneity in the host response to infection can be used (e.g., different healthy status, disease types, or clinical symptoms), as long as it can stratify samples from the same treatment group. Our data highlights the importance of heterogeneous response in the same disease model and provides a new perspective for many other disease studies.

In recent years, along with anti-influenza research of the gut microbiota unceasingly thorough, several strains have been confirmed to exert protective effects against influenza infection, including *Bifidobacterium longum* [20], *Lactobacillus casei* [21], *Lactobacillus rhamnosus* [22], and *Lactobacillus pentosus* [23]. However, because previous studies screened anti-influenza strains mainly by the exogenous administration of known probiotics, much important information is missed, which is crucial to further understand the anti-influenza effect of gut microbiota. For example, (1) whether these microbes exist in normal gut microbiota and exert anti-influenza effects without exogenous administration, (2) the dynamic characteristics of nearly all anti-influenza microbes are still unknown during influenza infection, and (3) whether there are systematic strategies to screen specific anti-influenza bacteria from the gut microbiota, instead of evaluating one by one from known probiotics, and so on. In the present research, to explain these key issues, we designed the LD_50_ approach as described above, which can be used for directional screening of anti-influenza strains from the whole gut microbiota, but not limited from known probiotics. We daily monitored the composition of gut microbiota from days 0 to 15 post-infection and successfully revealed the dynamic characteristics of the gut microbiota during influenza infection. What is more, we identified a novel anti-influenza gut microbe, *B. animalis*, and found that the host can enhance influenza resistance by expanding the gut population of endogenous *B. animalis* when lethal influenza infection occurs. Our data not only enriches our knowledge about anti-influenza effect of the gut microbiota, but also provides novel observations of the interaction between host and gut microbiota.

In the last several decades, notable developments were made in medical technology and equipment. However, many diseases remain difficult to completely control or cure because of the individual variability between patients. This challenge has become a very concerning and common problem that needs to be urgently resolved. To address this growing clinical demand, a great deal of effort worldwide has been invested into precision medicine, which considers individual variability and employs biomarkers to stratify patients, with the goal of improving the efficacy and safety of disease treatment [24]. However, because of the lack of reliable biomarkers, precision medicine is severely constrained with respect to the prevention and treatment of many diseases [25]. In fact, influenza infection, particularly severe influenza infection, remains associated with high mortality rates despite antiviral treatment and aggressive respiratory support [26]. In the current study, we showed that the *Bifidobacterium* genus was significantly enriched in the GX.SG group compared with the GX.DG group, and this enrichment was observed as early as 2 days post-infection (Additional file 15 Table S3). This observation suggests the existence of a bacterial predictor of secondary infection development. It is important to identify such early predictors of a patient’s prognosis/severity in humans. Our data indicated that *Bifidobacterium* has a potential as a novel biomarker for predicting the severity and mortality of influenza patients, which could thus contribute to further development of precision medicine strategies for influenza treatment.

*Bifidobacterium* are commensal microorganisms found in the gastrointestinal tract that can exert beneficial effects through various mechanisms including pathogen exclusion and immune modulation [27]. Here, we found that the protective effect of *B. animalis* against influenza was associated with changes in the lung and blood cytokine levels of infected mice, suggesting that *B. animalis* likely protects against influenza infection by modulating host immune response. To further understand the mechanism through which *B. animalis* protects the host against influenza infection, the functional metagenome comparison analysis was performed and implied several underlying functional molecules. Of which, we demonstrated that valine and CoA showed obvious anti-influenza effects in vivo. Notably, our data suggested that the anti-influenza effect of valine and CoA could be due to their immunoregulatory property or immune stimulation. This further supports our above speculation about the anti-influenza effect of *B. anim*alis. Although valine and CoA have been known to improve host health status or modulate immune responses [28–30], this is the first report about their anti-influenza effect, and further studies are still needed to explain their anti-influenza molecular mechanisms.

Some *Bifidobacterium* strains have been successfully applied in the food and pharmaceutical industries as probiotics. Indeed, we showed here that oral administration of *B. animalis* alone or the *B. animalis*/*B. pseudolongum* two-bacterium combination reduced the severity of the ensuing influenza infection. This might indicate that *B. animalis* may be developed into anti-influenza probiotics. However, some remaining questions still exist. For example, how soon before the infection the mice need to be gavaged, what is the duration of the probiotic effect, and whether there are other unknown effects that these bacteria strains could exert on the host when given as probiotics. Also, only one mouse strain was used in our research, whereas in some cases, the immune response in infection experiments is mouse strain-dependent, e.g., BALB/c mouse vs. C57BL/6 mouse. Lastly, we need to verify whether *B. animalis* still has similar anti-influenza effects in different hosts, especially in humans.

In addition to *Bifidobacterium*, other bacteria (e.g., *Lachnospiraceae_NK4A136_group*, *Lactobacillus*, and *Parasutterella*) whose abundance clearly decreased in the GX.DG group compared with the NC and GX.SG groups might also contribute to the host’s defense against influenza (Fig. 2d and Additional file 5: Fig S5). Indeed, *Lactobacillus* is recognized as a probiotic because of its health-promoting effects [31]. Certainly, additional studies are needed to verify their anti-influenza effect in detail. It is also intriguing that in the current study, some recognized probiotics showed a significant positive correlation with the severity of influenza infection. One such example is *Akkermansia*, which significantly increased in the GX.DG group at the later infection stage, but not in the NC and GX.SG groups. *Akkermansia muciniphila* exhibits a modulatory effect on the mucosal immune response in experiments involving a germ-free mouse and has the potential to be used as a next-generation beneficial microbe [32, 33]. However, our findings imply that *A. muciniphila* could exert an adverse effect during influenza infection, although we cannot exclude the possibility that *A. muciniphila* exerts a beneficial effect in other health scenarios.

## Conclusions

In conclusion, we successfully captured the heterogeneity of the response to influenza infection in the mouse model and identified *B. animalis* as a member of the gut microbiota that exerts a protective effect against influenza infection. We demonstrated that the gut population of endogenous *B. animalis* becomes more abundant upon influenza infection, thus protecting the host against the infection. Our study thus represents a novel example where the gut microbiota is able to protect hosts against an extraintestinal infection, possibly through new interactions between the host and gut microbiota. Our study also established the potential of using gut microbiota (e.g., *Bifidobacterium*) as a novel biomarker for predicting the severity and mortality of influenza patients, which could contribute to the development of precision medicine strategies for influenza treatment in future.

## Materials and methods

### Bacteria and virus

*B. pseudolongum* ATCC 25526 and *B. animalis* ATCC 25527 were obtained from the BeNa Culture Collection Co., Ltd. (China). These two strains were cultured in de Man-Rogosa-Sharpe medium (BD Difco, Le Pont de Claix, France) for 24 h under anaerobic conditions at 37 °C [34].

H7N9 virus strains MK453329 and MK453331 were isolated from chicken of China’s Guangxi and Hubei provinces in 2017, respectively, which were abbreviated as GX and HB. Of which, GX showed virulent in mice, but HB showed attenuated.

### Mouse infection for fecal analysis experiments and sample collection

Eight-week-old female C57BL/6 mice were used in all experiments. All animal experiments were performed in animal biosafety level 3 (ABSL3) conditions. SPF mice (*n* = 50, 18.72 ± 0.45) were ear-marked. Then, the mice were anesthetized by intraperitoneal injection of a mix of ketamine/xylazine (100 and 5 mg/kg, respectively) in 100 μL of sterile PBS [19] and were randomly selected to be intranasally infected with a LD_50_ dose of GX (*n* = 30) [equal to 500 50% embryo infective dose (EID_50_)] in 50 μL PBS, the same dose of HB (*n* = 10, 50 μL/mouse), or PBS (NC group) (*n* = 10, 50 μL/mouse). After infection, the body weight and survival of mice were monitored daily for 16 days (days 0 to 15 post-infection). Fecal samples were collected daily and immediately stored at − 80 °C for total DNA extraction and sequencing. In another repeat experiment, the lungs of mice were collected at day 10 post-infection, fixed in 4% formaldehyde, embedded in paraffin, and stained with hematoxylin and eosin (H&E) for histopathological analysis [35].

### Antibiotic treatment

SPF mice were treated with an ATB approach as previously described [18]. Briefly, ampicillin (1 mg/mL), streptomycin (5 mg/mL), vancomycin (0.25 mg/mL), and colistin (1 mg/mL) (Sigma-Aldrich) were added to the sterile drinking water of mice for 3 days. The ATB treatment was discontinued 1 day prior to FMT or *Bifidobacterium* colonization (see below).

### FMT experiments

To prepare the FMT inoculum, another 20 SPF mice were challenged with a LD_50_ of GX, and the feces were collected on days 3 and 9 post-infection, then were processed as previously described [36]. Briefly, within 2 h of collection, the fecal samples were homogenized in a sterile environment under N_2_ gas. The bacteria were collected by filtering through 0.25-mm stainless cell strainers and centrifuged at 6000×*g* for 15 min. The collected samples were resuspended in PBS with 10% (v/v) glycerol and frozen at − 80 °C. After 15 days of infection, the pre-collected fecal samples were divided into two groups based on whether the corresponding mice died or survived from the infection. Samples from the same group were pooled according to the collection time. Prior to FMT, the glycerol in the samples was replaced with PBS by centrifugation, and the number of live microbes was then counted by optical microscopy combined with methylene blue staining [37]. In addition, fecal samples from 10 PBS mock-infected mice were obtained and processed as described above. FMT was then performed by administering 100 μL of a suspension containing 1 × 10^8^ bacteria or PBS to ATB-pretreated SPF mice (*n* = 120, 20 mice per group) by oral gavage. One day later, all administered mice were intranasally challenged with a LD_50_ of GX, and their survival was monitored for 15 days post-infection.

### Gut colonization with commensal species

ATB-pretreated SPF mice or germ-free C57BL/6 mice were colonized by specific bacterial strains. Of which, germ-free mice were bred at the Department of Laboratory Animal Science in the Third Military Medical University in Chongqing, China. Before administration, *B. pseudolongum* and *B. animal*is were anaerobically cultured at 37 °C for 24 h then were harvested by centrifugation (5000 rpm, 5 min, 4 °C) and were resuspended in PBS with a concentration of 1 × 10^9^ colony-forming units (CFU)/mL, respectively. Eighty ATB-pretreated mice were randomly divided into four groups (*n* = 20/group) and received daily the following by oral gavage: vehicle (sterile PBS, 100 μL/mouse; group 1), a suspension of *B. pseudolongum* (100 μL/mouse, group 2), a suspension of *B. animalis* (100 μL/mouse, group 3), or a suspension of equal numbers of *B. pseudolongum* and *B. animalis* (100 μL/mouse, group 4). Three days later, all ATB-pretreated mice were intranasally challenged with a LD_50_ of GX, and their survival and body weight were monitored for 15 days post-infection. Similarly, 19 germ-free mice were randomly divided into groups 1 (*n* = 6), 3 (*n* = 6), and 4 (*n* = 7), which were administered the same treatments as the corresponding groups of ATB-pretreated mice. Three days later, all germ-free mice were infected with 0.5 × LD_50_ dose of the virulent H7N9 virus GX, and their survival rate was monitored for 15 days post-infection.

In another experiment, 60 ATB-pretreated mice were randomly divided into four groups of 15 mice (groups 1, 2, 3, and 4) and administered the corresponding treatments, as described above. On days 0, 3, and 5 post-infection, the blood and lungs from 5 mice from each group were collected. The lungs were homogenized to determine the virus titers and cytokine levels, and the serum was also prepared to determine cytokine levels (see below). For the histological examination, another 24 ATB-pretreated mice were randomly divided into four groups and were also treated as described above. On days 0 and 7 post-infection, 3 mice of each group were killed to collect the lungs.

### In vivo treatment of mice with specific metabolic molecules

To evaluate the anti-influenza effect of valine, isoleucine, lysine, or CoA, ATB-pretreated mice were gavaged daily for 1 week with 100 mg of valine, isoleucine, or lysine, or the same volume of PBS (200 μL), or were intraperitoneally injected daily for 3 days with 0.2 mg of CoA (Sigma-Aldrich) or the same volume of PBS (100 μL). For the survival rate and body weight curve, the mice were intranasally challenged with a LD_50_ of GX and monitored for 15 days post-infection (*n* = 10 mice per group). For the detection of virus titers and cytokine concentrations, 5 mice per group were randomly sacrificed for the collection of the lungs on day 0 and day 5 post-infection, respectively. For the histological examination, 3 mice per group were randomly sacrificed for the collection of the lungs on day 0 and day 7 post-infection.

### Analysis of cytokine concentrations

To determine the cytokine levels, the lungs were homogenized in PBS (1 mL/lung), and the homogenates were clarified by centrifugation. At the same time, the blood samples were allowed to clot at 4 °C overnight followed by centrifugation to separate the serum (1000 rpm, 4 °C, 5 min). The following cytokines were detected using Magnetic Luminex® Assay multiplex kits (R&D Systems): TNF-α, IL-1β, IL-6, IL-10, and IFN-γ. In addition, IFN-β levels in the lung homogenates and serum were determined using a VeriKine Mouse IFN-β enzyme-linked immunosorbent assay kit (BioLegend).

### Titration of the viral load in the lung

The detection of the virus in the lung was performed by inoculating 9–11-day-old SPF embryonated chicken eggs with the above-clarified lung homogenates. The inoculated eggs were incubated at 37 °C for 72 h, and the allantoic fluid was then harvested for hemagglutination assay [38]. The virus titers were calculated using the method described by Reed et al. [39].

### DNA extraction and barcoded sequencing of the 16S rRNA gene

Immediately after collection, all fecal samples were frozen at − 80 °C and transported to the laboratory on ice. The bacterial DNA from the fecal samples was extracted using a QIAamp DNA Stool Mini Kit (Qiagen, Germany). Briefly, add 1 mL InhibitEX Buffer to each stool sample. Vortex continuously for 1 min or until the stool sample is thoroughly homogenized. After homogenized, 600 μL supernatant was collected by centrifugation and pipetted into a new 2-mL microcentrifuge tube containing 25 μL proteinase K. Then, add 600 μL Buffer AL and vortex for 15 s. After incubated at 70 °C for 10 min, the lysate was added with 600 μL ethanol (96–100%). The mixed lysate was loaded on the QIAamp spin column by centrifugation. The QIAamp spin column was successively washed using 500 μL Buffer AW1 and 500 μL Buffer AW2. Finally, microbial DNA was eluted with 200 μL Buffer ATE and then stored at − 80 °C.

To characterize the taxonomic profile of the mouse gut microbiome, the V4 region of the 16S rRNA gene was sequenced using universal primers that target this region in most bacteria (F: 5′-GTGCCAGCMGCCGCGGTAA-3′; R: 5′-GGACTACHVGGGTWTCTAAT-3′; a 6-bp barcode unique to each sample was included in the reverse primer, R) [40]. Single amplifications were performed in 25-μL reactions with 50 ng of template DNA. The following polymerase chain reaction (PCR) conditions were used: 94 °C for 4 min, followed by 30 cycles of 94 °C for 30 s, 54 °C for 30 s, and 72 °C for 30 s, and, finally, 72 °C for 5 min. Normalized equimolar concentrations of the PCR products were then pooled and sequenced using the Illumina HiSeq2500 platform.

According to the specific barcodes, reads were assigned into different samples, and then the barcodes and sequencing primers were trimmed prior to assembly using perl script. Paired-end sequence reads were assembled using FLASH version 1.2.7 with default settings [41]. The sequencing data were quality-filtered using QIIME 1.9.1 (split_libraries_fastq.py) [42]. The data were next rarefied to the average number of reads detected in a single sample (84,598 reads). The trimmed sequences were filtered to remove chimeras and singletons and assigned to operational taxonomic units (OTUs) at a 16S rRNA sequence identify cutoff of 97%, using the UPARSE pipeline [43]. Representative sequences for each OTU were selected for classification using QIIME (assign_taxonomy.py) against the SILVA database (www.arb-silva.de; release 128) [44].

### Determination of alpha- and beta-diversity indices

The alpha- and beta-diversities of the microbiota dataset were analyzed using QIIME 1.9.1. The Shannon diversity index and the number of observed species per sample were used as the α-diversity metrics, and the β-diversity was calculated using the weighted UniFrac distances and assessed through PCoAs. The Bray-Curtis distances between samples were used for principal component analysis (prcomp function in R). For each grouping variable, 95% confidence ellipses were calculated using the vegan package.

### Metagenomic assembly, gene catalog generation, and rarefaction curve analysis

All samples were subjected to paired-end sequencing using the Illumina HiSeq 4000 platform (insert size of 350 bp and read length of 150 bp) by Novogene Bioinformatics Technology Co. Ltd. High-quality reads were obtained by filtering out low-quality reads with ambiguous “N” bases, adapter contamination, and *Mus musculus* genome (GRCm38.p6) DNA contamination from the Illumina raw reads. The alignment was performed using Bowtie 2 (version 2.2.4) with the following parameters: --end-to-end, --sensitive, -I 200, and -X 400 [45], and by removing reads with low-quality terminal bases.

The reads were subsequently de novo assembled into contigs using SOAP2 (version 2.04) and the following parameters: -d 1 -M 3 -R -u -F -K 55 [46]. Clean data were mapped against scaffolds using Bowtie 2, with the abovementioned parameters. The unused reads from each sample were then assembled using the same parameters. Open reading frames were predicted from the assembled contigs using Meta Gene Mark (prokaryotic GeneMark.hmm, version 2.10) [47]. A non-redundant gene catalog was then constructed using CD-HIT (version 4.5.8) and the following parameters: -G 0 -aS 0.9 -g 1 -d 0 -c 0.95 [48], and using a sequence identity threshold of 0.95 and a minimum coverage cutoff of 0.9. To determine the gene abundances, the reads were realigned with the gene catalog using Bowtie 2 and the following parameters: -m 200 -x 400 -s 119. Only genes with ≥ 2 mapped reads were deemed to be present in a sample [49]. The gene abundances were calculated by counting the number of reads and normalizing based on the gene length [50].

A rarefaction analysis was then performed to assess the richness of genes in various sample groups. For a given number of samples in the cohort, 100 random samplings were performed with replacement, and the total number of genes that could be identified in these samples was determined using R (version 2.15.3, vegan package).

### Taxonomic annotation and abundance profiling

For taxonomic assignment, all the predicted genes were aligned with the integrated non-redundant database (ftp://130.14.250.13/blast/db/FASTA/nr.gz, 20180118) using DIAMOND (version 0.9.9.110) and default parameters, with the exception of -k 50 -sensitive -e 0.00001 [51]. As previously described [49], significant matches for each gene (defined by *e* values ≤ 10 × *e* value of the top hit) and the retained matches were used to distinguish the taxonomic groups. The taxonomical level of each gene was determined by using the lowest common ancestor-based algorithm implemented in MEGAN [52]. The abundances of each taxonomic group were calculated by adding the abundances of genes annotated to a feature.

### Functional annotation

The constructed non-redundant gene catalog was aligned with functional databases, including the KEGG database (version 201801, www.kegg.jp/kegg), the EggNOG database (version 4.5, eggnogdb.embl.de/#/app/home), and the CAZy database (version 20150704, www.cazy.org), using DIAMOND (version 0.9.9.110) and default parameters, with the exception of -k 50 -sensitive -e 0.00001 [51]. Each protein was assigned to the database based on the highest scoring annotated hit(s) containing at least one high-scoring segment pairs (HSP) scoring over 60 [53]. The statistics of the relative abundances of different functional hierarchies were calculated by adding the relative abundances of genes annotated to the same feature.

### Co-abundance gene analysis and network of CAGs

To identify marker genes associated with a disease, genes that showed significant differences in relative abundance between any two groups were identified based on Benjamin-Hochberg *q* value < 0.05 (Wilcoxon rank-sum test)002C as described by Greenblum et al. [54]. The marker genes were then clustered according to the variation in abundance in all samples [55]. Clusters with more than 50 genes were identified as CAGs and used for further analysis. The CAG profiles were then determined by calculating the average gene depth signal weighted by the gene length.

A taxonomic assignment of CAGs was performed according to the taxonomy of tracer genes, as previously described [56]. Briefly, a CAG was assigned to a species only if 90% of the genes in the CAG were aligned with the species genome with 95% identity and 70% query overlap. A CAG was assigned to a genus only if 80% of the DNA (gene) and encoded protein sequences in the CAG aligned with 85% identity with genus data.

The enriched CAGs were identified according to Greenblum et al. [54]. These CAGs were clustered according to Spearman’s correlation. The co-occurrence network was visualized using Cytoscape (version 3.2.1). The enriched CAGs and/or genes were identified as described by Greenblum et al. [54]. Briefly, for each CAG, an OR score was calculated according to the abundance in the set of compared samples.

### Construction of a random forest model of CAGs

Using the random forest package in R, a random forest classifier was trained and tested using 80% and the remaining 20%, respectively, of the data obtained from the analysis of the CAG profiles. To evaluate the performance of the predictive model and improve the precision of cross-validation error curves, a 10-fold cross-validation using the training set was applied. The cross-validation error curves from five trials of the 10-fold cross-validation were averaged (10 test sets were included in each trial). The importance of each variable in the random forest models obtained using the full set of features was calculated based on the mean decrease in accuracy. The number of variables with the lowest cross-validation error was 1000. Consequently, the predictive model was constructed using the 1000 most important variables and further applied for ROC analysis. The performance of each smaller model was determined as the AUC obtained using the test set, and the confidence intervals for ROC curves were calculated using the randomForest and pROC R package.

### qRT-PCR

According to the above challenge procedure, SPF mice were randomly intranasally infected with a LD_50_ dose of influenza virus GX (*n* = 20) or equivoluminal PBS (NC group, *n* = 10). At days 2 and 10 post-infection, fecal samples were collected, and the DNA was extracted respectively. After 15 days of infection, the pre-extracted DNA of fecal samples were divided into two groups based on whether the corresponding mice died or survived from the infection. Then, qRT-PCR was performed to detect *B. pseudolongum* and *B. animalis* as previously described [57]. Briefly, 2 ng of fecal DNA was amplified with SYBR Green (Roche) and primers B_plon-f/r or B_ani-f/r to detect *B. pseudolongum* or *B. animal*is, respectively [58]. To draw a standard curve, the PCR blunt vector (Invitrogen) was used, which contains a single copy of the 16S rRNA gene of *B. pseudolongum* ATCC 25526 or *B. animalis* ATCC 25527. The copy numbers of *B. pseudolongum* or *B. animalis* were calculated according to the standard curve.

### Statistical analysis

The R packages stats and vegan were used for the statistical analyses. ANOSIM and Adonis analyses were performed using the vegan package in R software (version 2.15.3). A LDA at various taxonomic levels (biomarkers from the phylum to the genus levels) and a LEfSe analysis with various parameters (using a logarithmic LDA score threshold of 4.0) were performed to identify the taxonomic biomarkers that characterize the differences between mice experiencing different fates (i.e., survival or death after infection) [59]. Analyses of the microbial communities showing different abundances between groups were conducted at various levels (i.e., the phylum, class, order, family, genus, and species) using Metastats [60]. Data were analyzed based on the *P* value obtained using Benjamini-Hochberg false discovery rate correction [function p. adjust in the stats package of R (version 3.1.1)] and adjusted using the Metastats software R (version 3.1.1).

## Supplementary information

**Additional file 1: Fig S1.** The lesions of lung in the GX.DG mice were notably more severe than those in the GX.SG mice. Experimental description references to Fig. 1c. All experiments were performed at least twice under similar conditions and yielded similar results.

**Additional file 2: Fig S2.** α-Diversity analysis (Shannon index) among the GX.DG, GX.SG, HB, and NC groups. Experimental description references to Fig. 2.

**Additional file 3: Fig S3.** Principal coordinate analysis (PCoA) of the weighted UniFrac distances among the GX.DG, GX.SG, HB, and NC groups at four infection stages. Experimental description references to Fig. 2. Sample clustering at stage 4 (days 9-15) revealed differences between the GX.DG group and the other three groups.

**Additional file 4: Fig S4.** Bacterial phyla showing significant differences in abundance, other than those shown in Fig. 2c. Experimental description references to Fig. 2. The data are presented as the relative abundance of specific microbiota at the phylum level in indicated groups and were analyzed by Wilcoxon-Mann-Whitney test, at *P<0.05, **P<0.01, and ***P<0.001.

**Additional file 5: Fig S5.** Classification of bacterial genera based on their variation trends in the four mouse groups. Experimental description references to Fig. 2. **(A)** Bacteria whose abundance increased only in the GX.DG group. **(B)** Bacteria whose abundance first increased and then decreased only in the GX.DG group. **(C)** Bacteria whose abundance increased in both the GX.DG and GX.SG groups. **(D)** Bacteria whose abundance decreased only in the GX.DG group. **(E)** Bacteria whose abundance increased in the GX.DG, GX.SG, and HB groups, with the greatest increase in the HB group.

**Additional file 6 Fig S6.** Rarefaction curves. The rarefaction analysis involved random sampling with replacement and estimation of the total number of genes that were identified in the samples. The values represent that the curve in all group is near smooth when the sample number are great enough with few new genes detected.

**Additional file 7: Table S1.** Detailed information for the 98 annotated co-abundance groups.

**Additional file 8: Fig S7.** LDA effect size (LEfSe) comparison of gut microbes at species level in the GX.SG and GX.DG groups. Experimental description references to Fig. 3. **(A–E)** LEfSe analysis of gut microbes collected on days 5 **(A)**, 8 **(B)**, 9 **(C)**, 10 **(D)**, and 11 **(E)** post-infection.

**Additional file 9: Fig S8.** Copy numbers of *B. pseudolongum* and *B. animalis* in feces. **(A)** Survival rate of mice infected with a LD_50_ dose of GX virus (*n*=20) or equivoluminal PBS (NC group, *n*=10). **(B and C)** On day 2 and 10 post-infection, fecal samples were collected, and the DNA was extracted respectively. After 15 days infection, the pre-extracted DNA of fecal samples were divided into two groups based on whether the corresponding mice died or survived from the infection. Then, qRT-PCR was performed to detect the copy numbers of *B. pseudolongum***(B)** and *B. animalis***(C)**. The data are presented as the mean ± SD. Statistics for the copy number was two-way ANOVA. *P<0.05, **P<0.01. All experiments were performed at least twice under similar conditions and yielded similar results.

**Additional file 10: Fig S9.** Cytokine concentrations in the mouse blood. Before infection, the ATB-pretreated mice were administered with *B. pseudolongum* alone, *B. animalis* alone, the two-bacterium combination, or PBS, with 24 mice in each group. Blood samples were respectively collected on days 0, 3, and 5 post-infection (*n*=8 mice/group) for the determination of cytokine concentrations. The data are presented as the mean ± SD. *P<0.05 and **P<0.01 (two-way ANOVA). All experiments were performed at least twice under similar conditions and yielded similar results.

**Additional file 11: Table S2.** Detailed information for the 2278 KEGG orthology terms enriched in the GX.SG group compared with the GX.DG or NC groups.

**Additional file 12: Fig S10.** Functional enrichment in the GX.SG group compared with the GX.DG or NC group. The functions enriched in the GX.SG group are displayed according to the infection time. The asterisks indicate significant functional enrichment in the GX.SG group compared with the GX.DG or NC group (*P<0.05 and **P<0.01). Blank represents no significant functional enrichment in the GX.SG group compared with the GX.DG or NC group.

**Additional file 13: Fig S11.** Viral loads detection in A549 cells. A549 cells were incubated for 12 hours with valine or CoA, and then infected with H7N9 influenza virus GX at a multiplicity of infection (MOI) of 0.01 50% embryo infective dose (EID_50_). After 24 hours, viral RNA was extracted from the culture supernatant. Viral load is expressed as viral RNA copies (NP gene) (mean ± SD).

**Additional file 14: Fig S12.** Relative abundance of the *Bifidobacterium* genus in the NC and GX groups. The merging of the GX.DG and GX.SG sample data shown in Fig. 2d into a single group (GX) almost completely eliminated the differences in *Bifidobacterium* abundance between the NC and GX groups.

**Additional file 15: Table S3.** Significance analysis of the relative abundance of *Bifidobacterium* genus between different groups, before and after merging GX.SG and GX.DG into GX.

**Additional file 16.** Review history.

## Data Availability

16S rRNA gene sequencing data and Metagenomic sequencing data for all samples used in this study are available under the European Nucleotide Archive (ENA) project PRJEB31298 [61].
