# Supplementary figures and images for: Influenza infection elicits an expansion of gut population of endogenous Bifidobacterium animalis which protects mice against infection
Source: Genome Biol. 2020 Apr 28;21:99. doi: 10.1186/s13059-020-02007-1 (PMC7187530; doi:10.1186/s13059-020-02007-1)

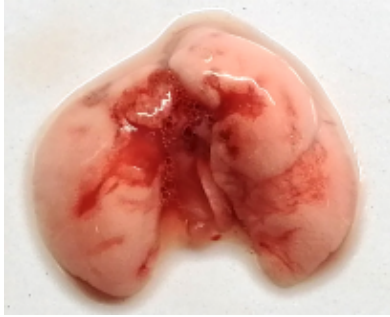

**NC**

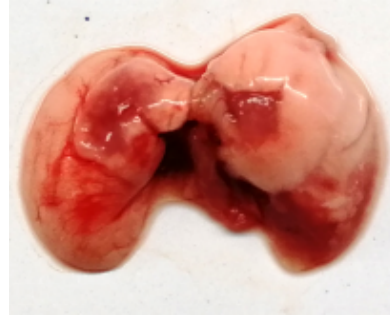

**HB**

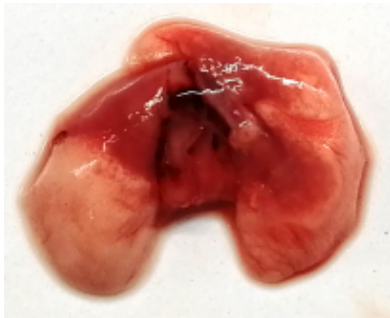

**GX.SG**

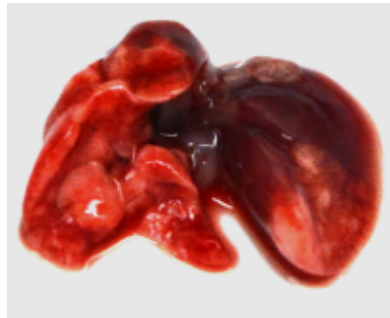

**GX.DG**

Supplement: Supplementary file 1 — Additional file 1: Fig S1. The lesions of lung in the GX.DG mice were notably more severe than those in the GX.SG mice. Experimental description references to Fig. 1c. All experiments were performed at least twice under similar conditions and yielded similar results. [file 13059_2020_2007_MOESM1_ESM.pdf]

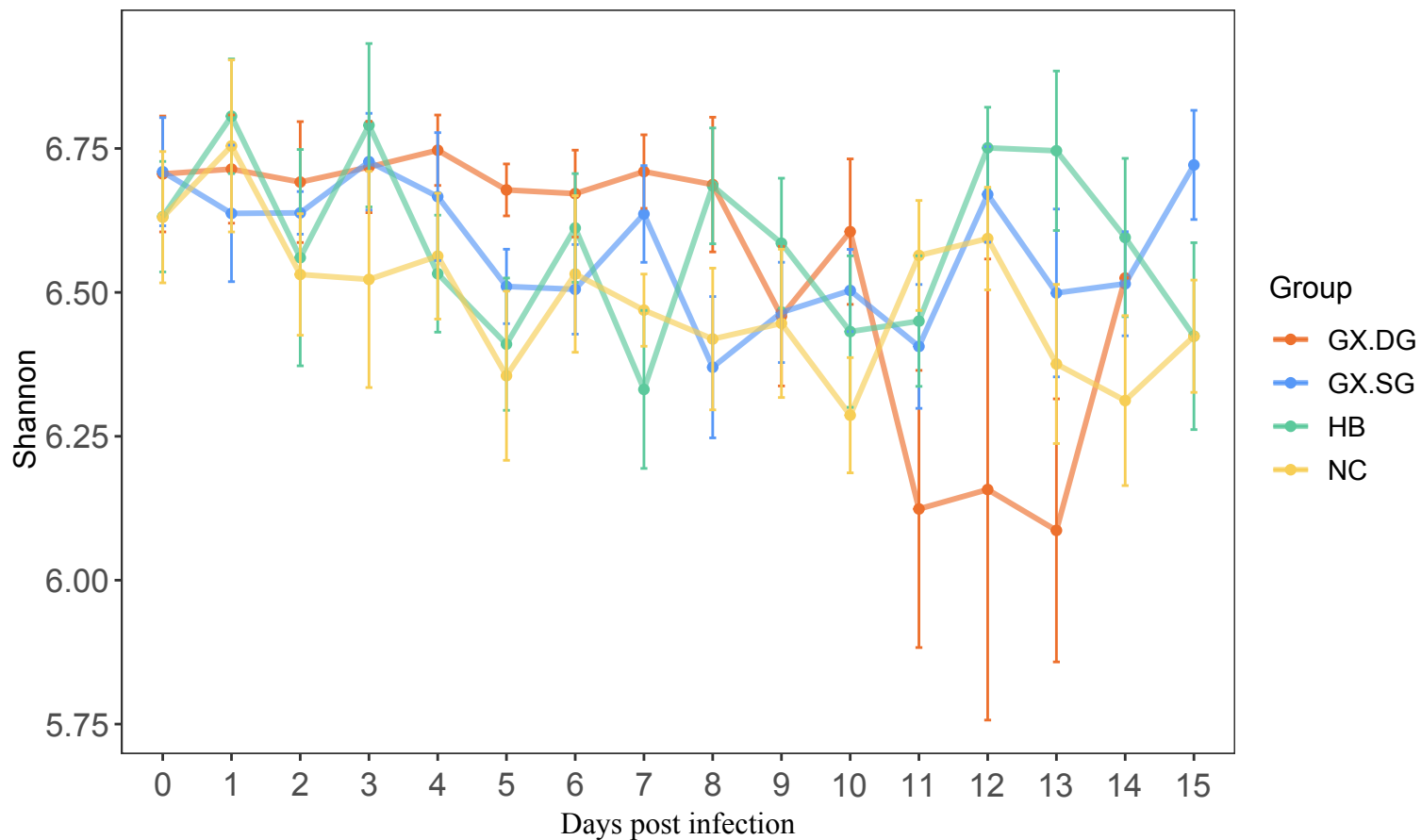

Supplement: Supplementary file 2 — Additional file 2: Fig S2. α-Diversity analysis (Shannon index) among the GX.DG, GX.SG, HB, and NC groups. Experimental description references to Fig. 2. [file 13059_2020_2007_MOESM2_ESM.pdf]

Day 0 post infection

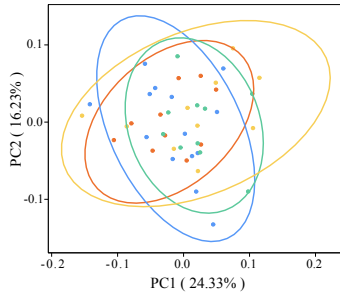

Days 1-4 post infection

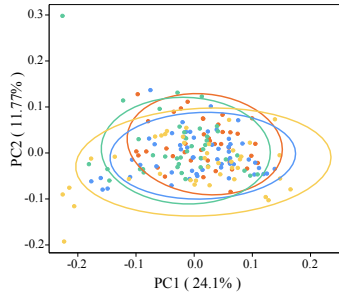

Days 5-8 post infection

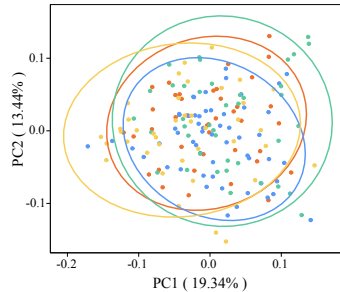

Days 9-15 post infection

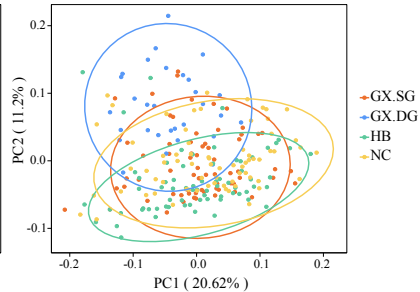

Supplement: Supplementary file 3 — Additional file 3: Fig S3. Principal coordinate analysis (PCoA) of the weighted UniFrac distances among the GX.DG, GX.SG, HB, and NC groups at four infection stages. Experimental description references to Fig. 2. Sample clustering at stage 4 (days 9-15) revealed differences between the GX.DG group and the other three groups. [file 13059_2020_2007_MOESM3_ESM.pdf]

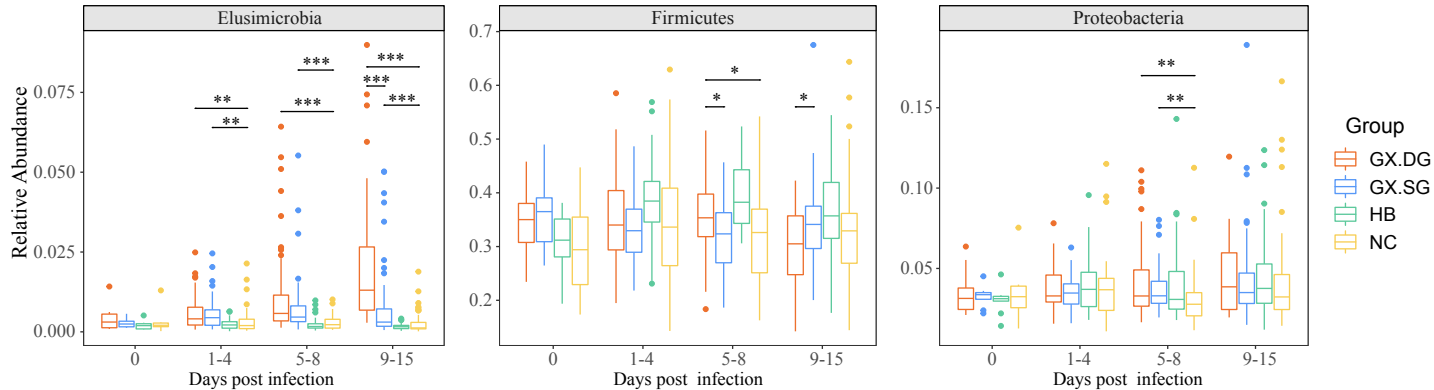

Supplement: Supplementary file 4 — Additional file 4: Fig S4. Bacterial phyla showing significant differences in abundance, other than those shown in Fig. 2c. Experimental description references to Fig. 2. The data are presented as the relative abundance of specific microbiota at the phylum level in indicated groups and were analyzed by Wilcoxon-Mann-Whitney test, at *P<0.05, **P<0.01, and ***P<0.001. [file 13059_2020_2007_MOESM4_ESM.pdf]

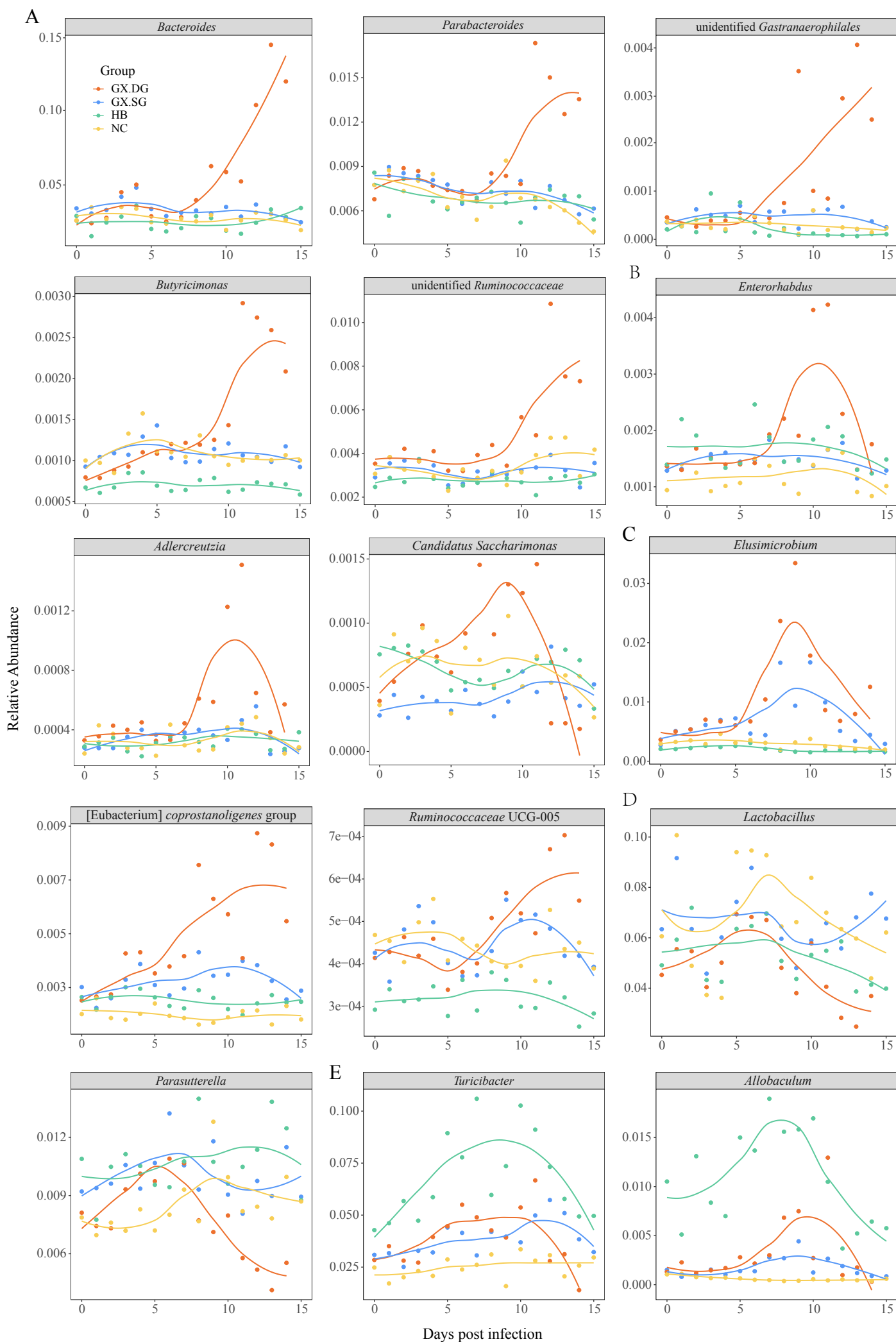

Supplement: Supplementary file 5 — Additional file 5: Fig S5. Classification of bacterial genera based on their variation trends in the four mouse groups. Experimental description references to Fig. 2. (A) Bacteria whose abundance increased only in the GX.DG group. (B) Bacteria whose abundance first increased and then decreased only in the GX.DG group. (C) Bacteria whose abundance increased in both the GX.DG and GX.SG groups. (D) Bacteria whose abundance decreased only in the GX.DG group. (E) Bacteria whose abundance increased in the GX.DG, GX.SG, and HB groups, with the greatest increase in the HB group. [file 13059_2020_2007_MOESM5_ESM.pdf]

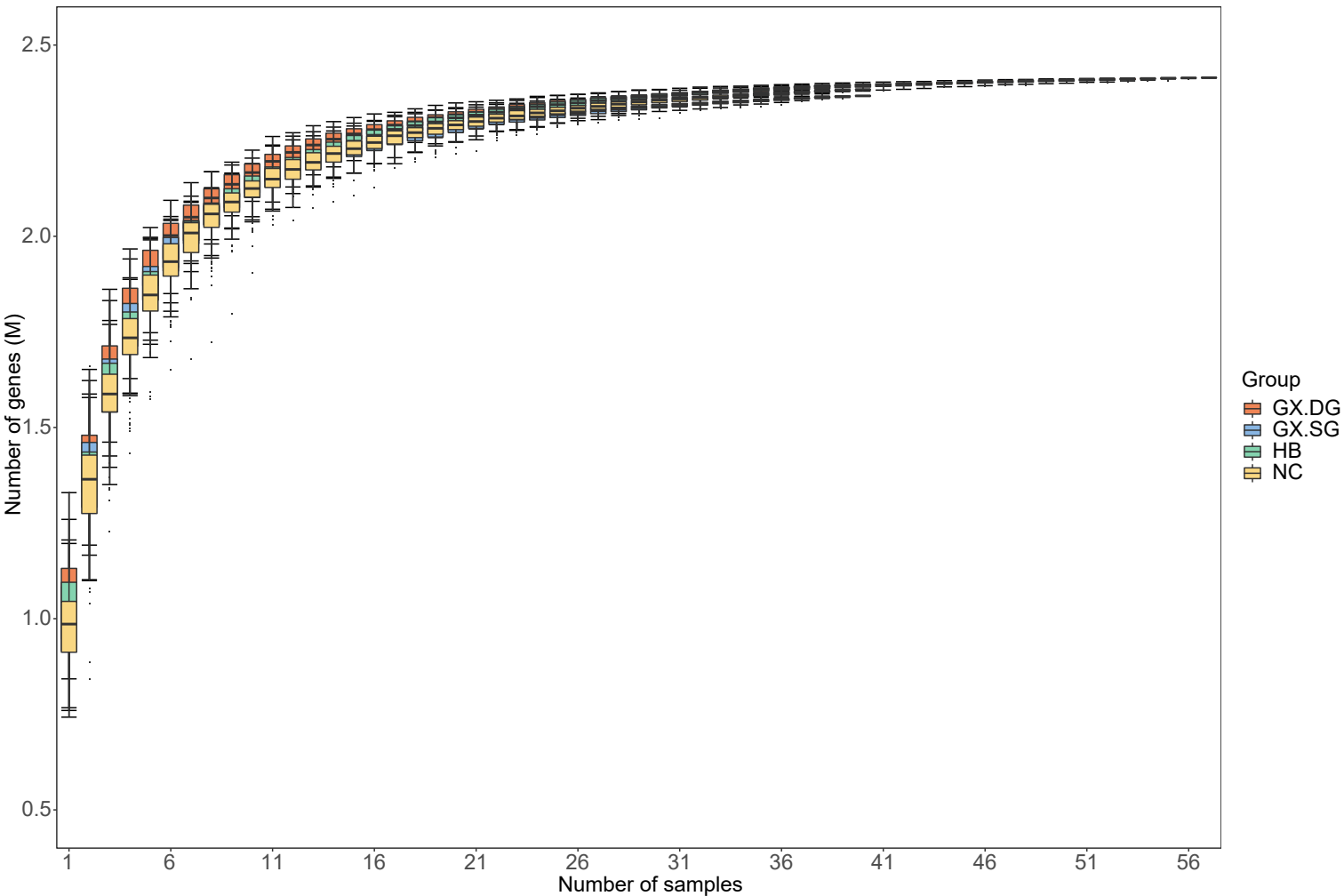

Supplement: Supplementary file 6 — Additional file 6 Fig S6. Rarefaction curves. The rarefaction analysis involved random sampling with replacement and estimation of the total number of genes that were identified in the samples. The values represent that the curve in all group is near smooth when the sample number are great enough with few new genes detected. [file 13059_2020_2007_MOESM6_ESM.pdf]

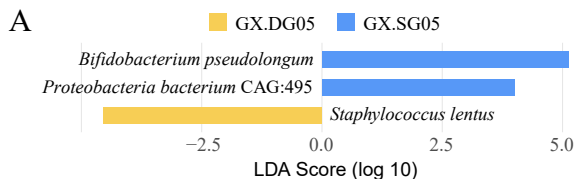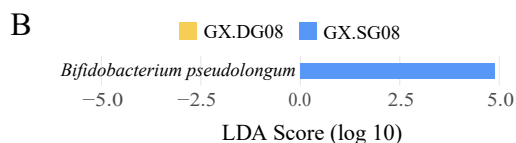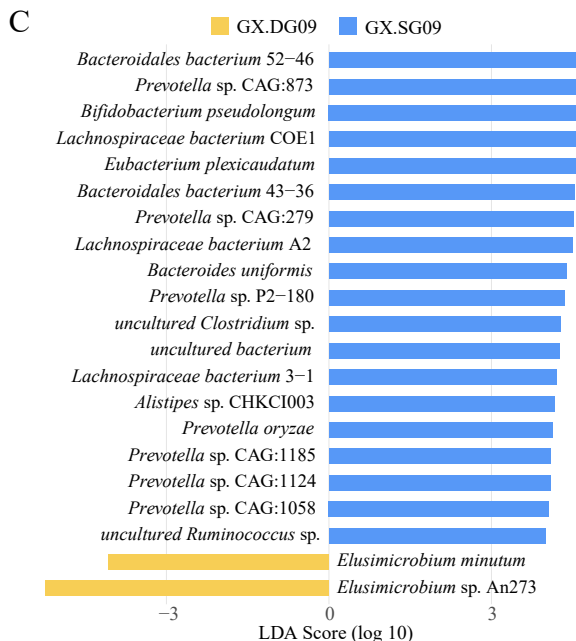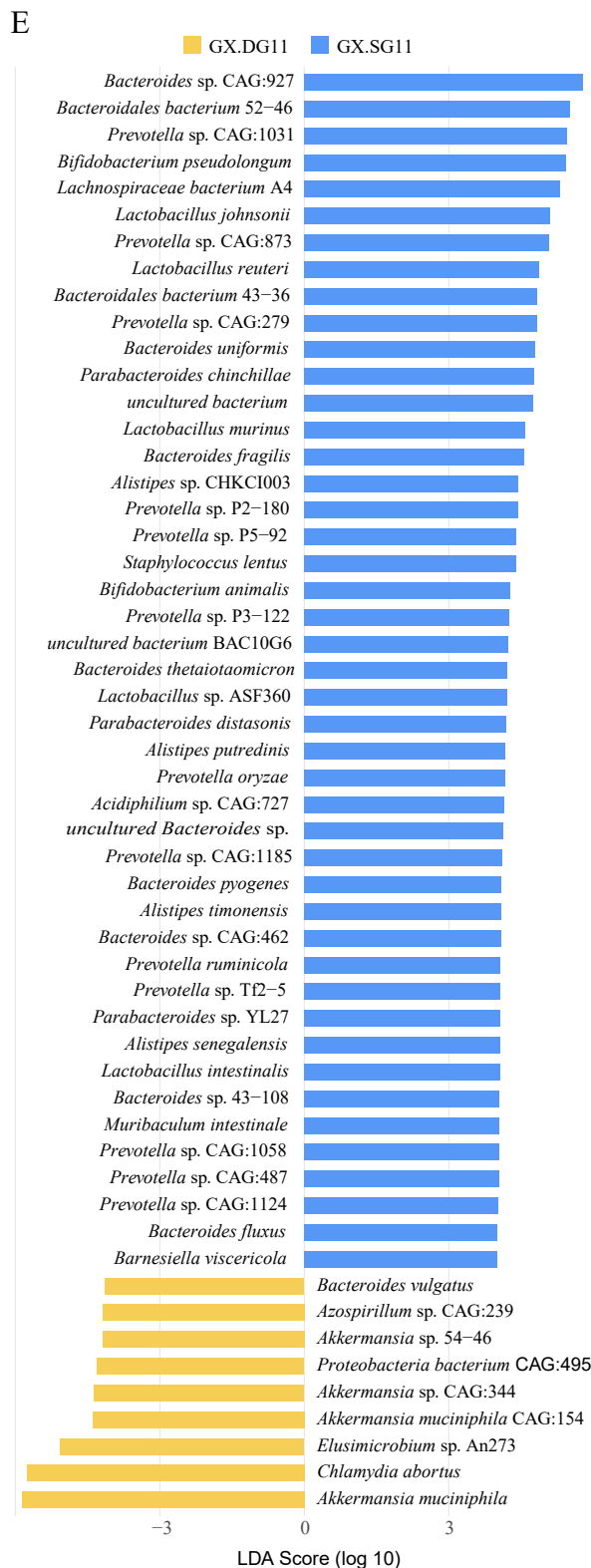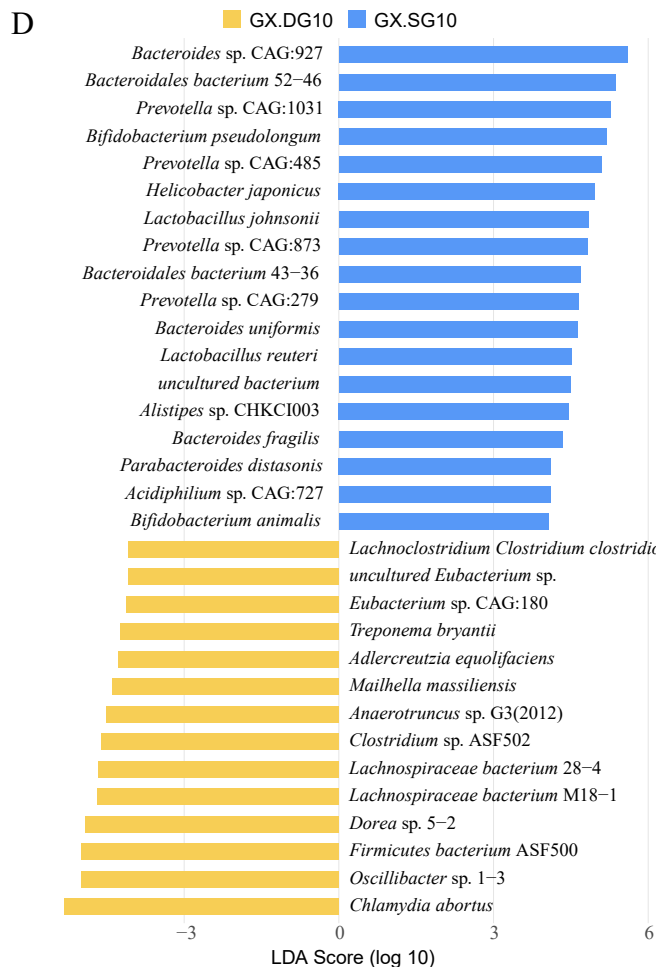

Supplement: Supplementary file 8 — Additional file 8: Fig S7. LDA effect size (LEfSe) comparison of gut microbes at species level in the GX.SG and GX.DG groups. Experimental description references to Fig. 3. (A–E) LEfSe analysis of gut microbes collected on days 5 (A), 8 (B), 9 (C), 10 (D), and 11 (E) post-infection. [file 13059_2020_2007_MOESM8_ESM.pdf]

**A**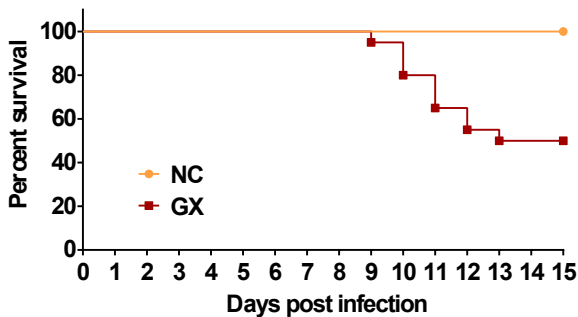**B**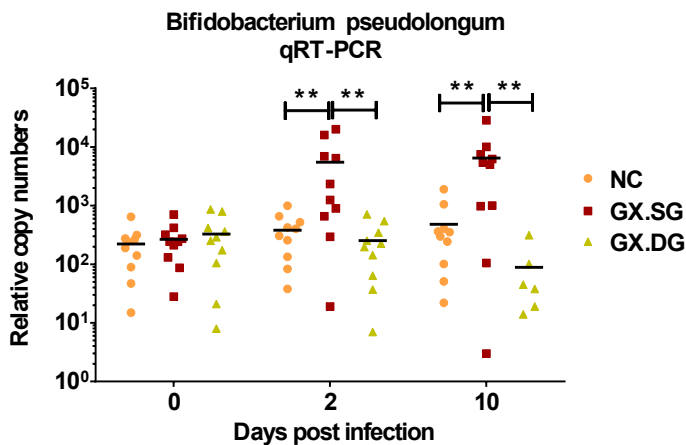**C**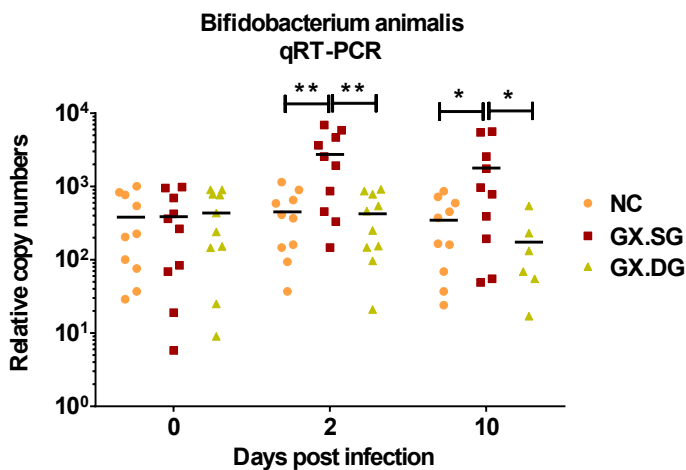

Supplement: Supplementary file 9 — Additional file 9: Fig S8. Copy numbers of B. pseudolongum and B. animalis in feces. (A) Survival rate of mice infected with a LD50 dose of GX virus (n=20) or equivoluminal PBS (NC group, n=10). (B and C) On day 2 and 10 post-infection, fecal samples were collected, and the DNA was extracted respectively. After 15 days infection, the pre-extracted DNA of fecal samples were divided into two groups based on whether the corresponding mice died or survived from the infection. Then, qRT-PCR was performed to detect the copy numbers of B. pseudolongum(B) and B. animalis(C). The data are presented as the mean ± SD. Statistics for the copy number was two-way ANOVA. *P<0.05, **P<0.01. All experiments were performed at least twice under similar conditions and yielded similar results. [file 13059_2020_2007_MOESM9_ESM.pdf]

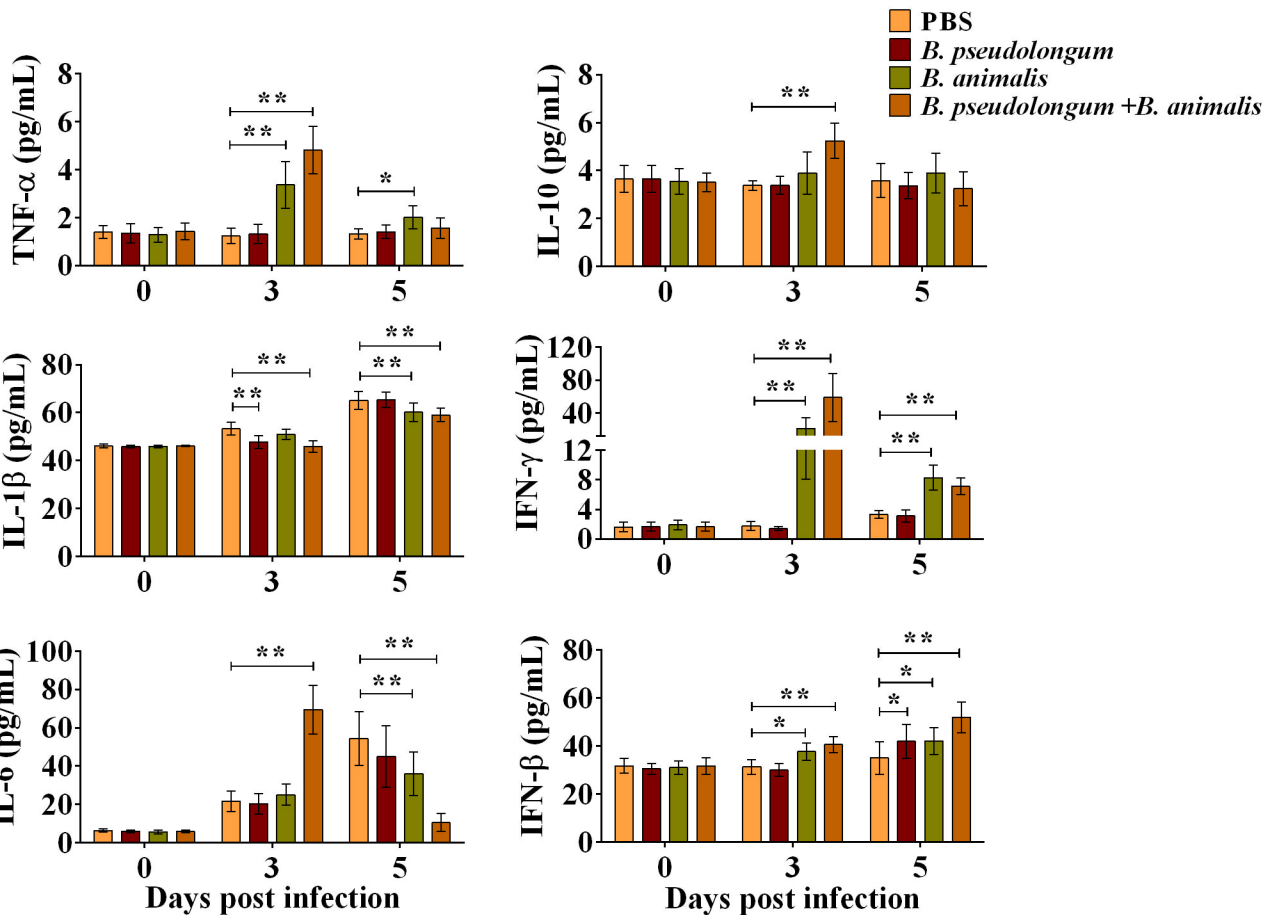

Supplement: Supplementary file 10 — Additional file 10: Fig S9. Cytokine concentrations in the mouse blood. Before infection, the ATB-pretreated mice were administered with B. pseudolongum alone, B. animalis alone, the two-bacterium combination, or PBS, with 24 mice in each group. Blood samples were respectively collected on days 0, 3, and 5 post-infection (n=8 mice/group) for the determination of cytokine concentrations. The data are presented as the mean ± SD. *P<0.05 and **P<0.01 (two-way ANOVA). All experiments were performed at least twice under similar conditions and yielded similar results. [file 13059_2020_2007_MOESM10_ESM.pdf]

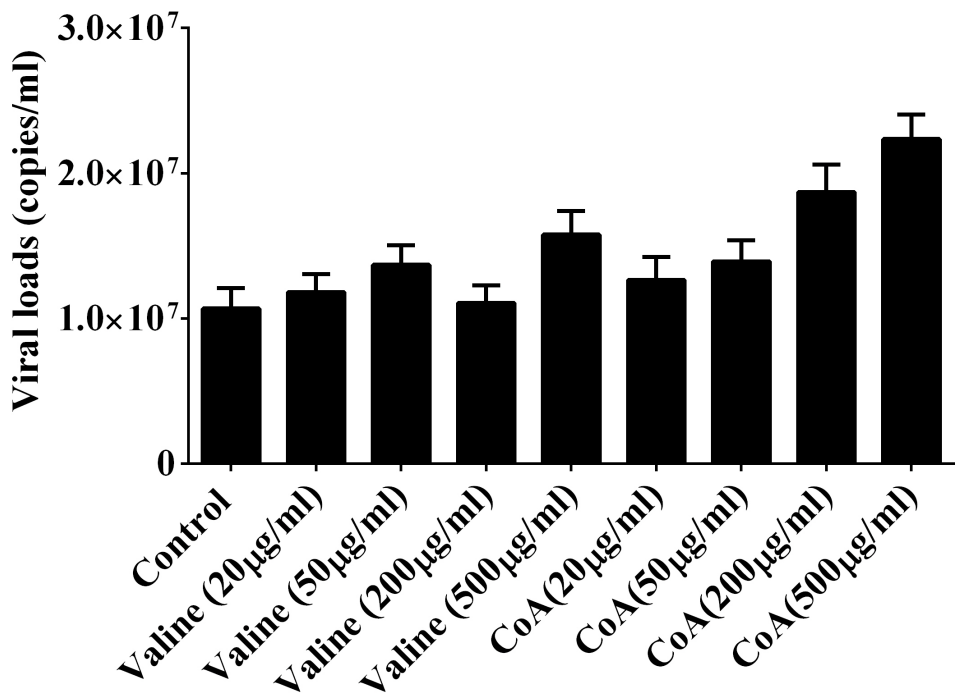

Supplement: Supplementary file 13 — Additional file 13: Fig S11. Viral loads detection in A549 cells. A549 cells were incubated for 12 hours with valine or CoA, and then infected with H7N9 influenza virus GX at a multiplicity of infection (MOI) of 0.01 50% embryo infective dose (EID50). After 24 hours, viral RNA was extracted from the culture supernatant. Viral load is expressed as viral RNA copies (NP gene) (mean ± SD). [file 13059_2020_2007_MOESM13_ESM.pdf]

*Bifidobacterium*

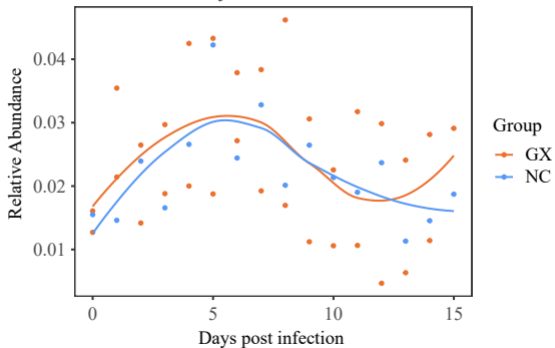

Supplement: Supplementary file 14 — Additional file 14: Fig S12. Relative abundance of the Bifidobacterium genus in the NC and GX groups. The merging of the GX.DG and GX.SG sample data shown in Fig. 2d into a single group (GX) almost completely eliminated the differences in Bifidobacterium abundance between the NC and GX groups. [file 13059_2020_2007_MOESM14_ESM.pdf]
