# Supplementary material for: Influenza infection elicits an expansion of gut population of endogenous Bifidobacterium animalis which protects mice against infection
Source: Genome Biol. 2020 Apr 28;21:99. doi: 10.1186/s13059-020-02007-1 (PMC7187530; doi:10.1186/s13059-020-02007-1)
Supplement: Supplementary file 12 — Additional file 12: Fig S10. Functional enrichment in the GX.SG group compared with the GX.DG or NC group. The functions enriched in the GX.SG group are displayed according to the infection time. The asterisks indicate significant functional enrichment in the GX.SG group compared with the GX.DG or NC group (*P<0.05 and **P<0.01). Blank represents no significant functional enrichment in the GX.SG group compared with the GX.DG or NC group. [file 13059_2020_2007_MOESM12_ESM.pdf]

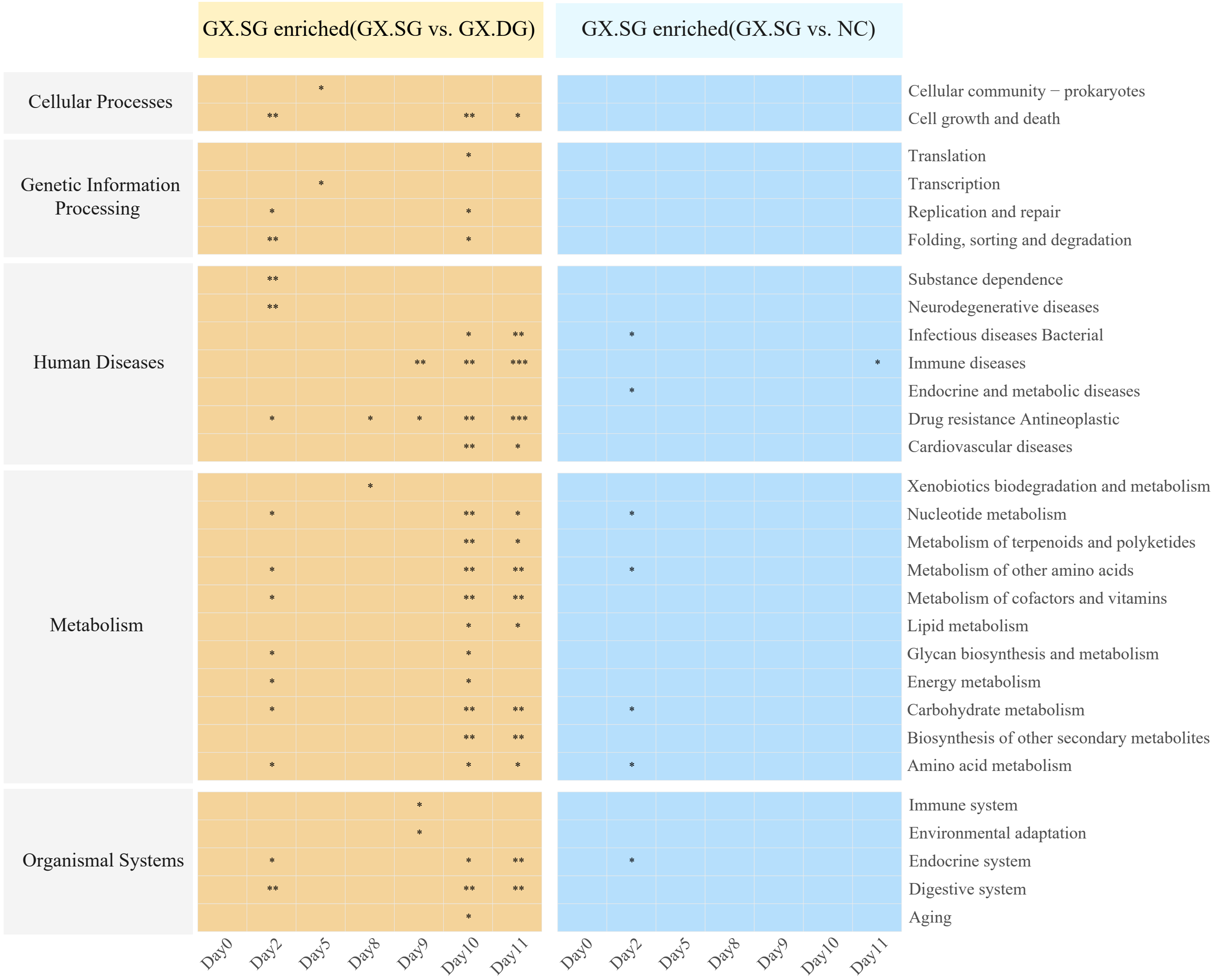
GX.SG enriched(GX.SG vs. GX.DG)
GX.SG enriched(GX.SG vs. NC)
Cellular Processes
Genetic Information Processing
Human Diseases
Metabolism
Organismal Systems
Cellular community − prokaryotes
Cell growth and death
Translation
Transcription
Replication and repair
Folding, sorting and degradation
Substance dependence
Neurodegenerative diseases
Infectious diseases Bacterial
Immune diseases
Endocrine and metabolic diseases
Drug resistance Antineoplastic
Cardiovascular diseases
Xenobiotics biodegradation and metabolism
Nucleotide metabolism
Metabolism of terpenoids and polyketides
Metabolism of other amino acids
Metabolism of cofactors and vitamins
Lipid metabolism
Glycan biosynthesis and metabolism
Energy metabolism
Carbohydrate metabolism
Biosynthesis of other secondary metabolites
Amino acid metabolism
Immune system
Environmental adaptation
Endocrine system
Digestive system
Aging
Day0
Day2
Day5
Day8
Day9
Day10
Day11
Day0
Day2
Day5
Day8
Day9
Day10
Day11
