# Supplementary material for: Influenza infection elicits an expansion of gut population of endogenous Bifidobacterium animalis which protects mice against infection
Source: Genome Biol. 2020 Apr 28;21:99. doi: 10.1186/s13059-020-02007-1 (PMC7187530; doi:10.1186/s13059-020-02007-1)
Supplement: Supplementary file 15 — Additional file 15: Table S3. Significance analysis of the relative abundance of Bifidobacterium genus between different groups, before and after merging GX.SG and GX.DG into GX. [file 13059_2020_2007_MOESM15_ESM.docx]

**Table S3:** Significance analysis of the relative abundance of *Bifidobacterium* genus between different groups, before and after merging GX.SG and GX.DG into GX

| Days post infection | *P*-value (Student's t-test) | | | |
| --- | --- | --- | --- | --- |
|  | Before mergeing GX group | | | After mergeing GX group |
|  | GX.DG vs.GX.SG | GX.DG vs. NC | GX.SG vs.NC | GX vs. NC |
| 0 | 0.6064 | 0.5412 | 0.8938 | 0.7086 |
| 1 | 0.2764 | 0.2948 | 0.0448 | 0.1211 |
| 2 | 0.0130 | 0.1285 | 0.7420 | 0.4151 |
| 3 | 0.2851 | 0.8172 | 0.1125 | 0.4218 |
| 4 | 0.0513 | 0.4286 | 0.2088 | 0.7622 |
| 5 | 0.0465 | 0.0188 | 0.9442 | 0.2179 |
| 6 | 0.2698 | 0.7397 | 0.1716 | 0.3789 |
| 7 | 0.0322 | 0.1322 | 0.6482 | 0.5300 |
| 8 | 0.2504 | 0.6629 | 0.3143 | 0.6029 |
| 9 | 0.0568 | 0.0318 | 0.7466 | 0.4454 |
| 10 | 0.1139 | 0.0833 | 0.8806 | 0.5151 |
| 11 | 0.1759 | 0.3189 | 0.2310 | 0.4743 |
| 12 | 0.2226 | 0.2377 | 0.5656 | 0.8460 |
| 13 | 0.1977 | 0.4054 | 0.0484 | 0.1123 |
| 14 |  |  | 0.0007 | 0.0030 |
| 15 |  |  | 0.2723 | 0.2723 |
