# Supplementary material for: Influenza infection elicits an expansion of gut population of endogenous Bifidobacterium animalis which protects mice against infection
Source: Genome Biol. 2020 Apr 28;21:99. doi: 10.1186/s13059-020-02007-1 (PMC7187530; doi:10.1186/s13059-020-02007-1)
Supplement: Supplementary file 16 — Additional file 16. Review history. [file 13059_2020_2007_MOESM16_ESM.docx]

Review History

**First round of review**

**Reviewer 1**

**Are you able to assess all statistics in the manuscript, including the appropriateness of statistical tests used?** No, I do not feel adequately qualified to assess the statistics.

**Comments to author:**

In this paper, Zhang and colleagues aimed at investigating the role of the gut microbiome in the response to influenza. For this purpose, the authors used an approach based on the fecal microbiotra transfer from influenza-resistant mice to recipient mice and showed some protective effect against influenza infection. This protective effect could be recapitulated by Bifidobacterium animalis alone.

Major comments:
-     The authors describe intestinal lesions in mice that died from influenza infection. The histology shown in Fig1D does not support this conclusion. Moreover, if any lesions are present, it might be due to the global circulation impairment in this severely infected mice.
-     The authors showed that fecal microbiota transfer using stool from mice at 3 or 9 days after infection had some effects on susceptibility to infection of recipient mice (microbiota of mice that will die from infection worsen the severity in recipients). Although this experiment is interesting, it is very difficult to analyze as the stools used for FMT come from mice already infected by influenza. It is thus likely that the worsening or protective effect is due to the reaction of the microbiota to the infection and not to a basal microbiota state. In the same way, the results of the control groups are difficult to interpret. It would be much easier to perfom FMT with baseline (before infection) stool.
-     All the results regarding the potential mechanism of action are speculative. Experiemnetal validation would be needed to validate it.
-     In figure 4,  the mice are treated by antibiotics. So th effect of the tested Bifidobacterium might not be specific. It would be more relevant to supplement non antibiotics treated mice with Bifidobacterium on a daily basis to evaluate the effect
-     There are many strains of B animalis. Why this specific one has been chosen? It would have been more relevant to work with the one isolated from the animal facility.
-     DNA extraction method should be described more precisely. Was there a mechanical lysis step?

**Reviewer 2**

**Are you able to assess all statistics in the manuscript, including the appropriateness of statistical tests used?** No, I do not feel adequately qualified to assess the statistics.

**Comments to author:**

In the manuscript, entitled "Influenza infection elicits an expansion of gut population of endogenous Bifidobacterium animalis which protects the host against infection", authors identified gut commensal microbes that are responsible for anti-influenza protection and verified their effects in defined animal models. Experimental procedures are robustly provided and microbial population analysis was well performed. Authors also proposed a potential mechanism of protection by functional metagenomic analysis. Overall, results are of interest in that changes in commensal microbial population induced their host to be more resistant to viral infection. However, presented results are still at the preliminary stage, and further additional results should be provided to corroborate authors' claims.

1.     Based on Fig. 1A, GX-infected mice begun to die at 9 days post-infection. How could authors collect feces from GX.DG donor at 3 day post-infection as illustrated in Fig. 1E?
2.     Authors performed co-abundance genes (CAGs) analysis to provide microbial population at the species level. Since Bifidobacterium animalis is culturable, it needs to be shown that Bifidobacterium animalis population is actually enriched in GX.SG vs. GX.DG. Authors can try to cultivate fecal matters discharged from GX.SG and GX.DG groups and measure changes in B. animalis CFUs on a Bifidobacterium selective agar plates. Alternatively, quantitative RT-PCR can be performed to compare the copy-number of B. animalis-specific genes.
3.     Influenza virus is infected via intranasal route. FMT affects microbial population changes in the gut. Additional experiments are necessary to evaluate events that occurred through this gut-airway axis. Images shown in Fig. 4F do NOT clearly show that improved lung pathology in mice administered with B. animalis alone or with both.  
4.     Authors claimed that B. animalis-mediated anti-viral effects are due to the biosynthesis of valine, isoleucine, lysine and coenzyme A. But, no supporting evidence is provided. To be able to claim that argument, authors need to perform additional experiments. For example, these molecules are chemically provided into the mouse airway and test the effects. In addition, authors need to provide reasonable explanation how these molecules can be delivered to the airway or if not, how these molecules strengthen the anti-viral responses.

**Authors Response**

**Point-by-point responses to the reviewers’ comments:**

Reviewer #1: In this paper, Zhang and colleagues aimed at investigating the role of the gut microbiome in the response to influenza. For this purpose, the authors used an approach based on the fecal microbiotra transfer from influenza-resistant mice to recipient mice and showed some protective effect against influenza infection. This protective effect could be recapitulated by Bifidobacterium animalis alone.

**Response: Special thanks to you for your good comments. As your suggestion, all amendments are highlighted in red in the revised manuscript. At the same time, the responses for your comments have also been listed below.**

Major comments: -The authors describe intestinal lesions in mice that died from influenza infection. The histology shown in Fig1D does not support this conclusion. Moreover, if any lesions are present, it might be due to the global circulation impairment in these severely infected mice.

**Response: Thank you for your important comments. Our data showed that some disintegrated intestinal epithelial cells were observed in the caeca of GX.DG, but not in other groups. Considering that gastrointestinal disorder is one of common symptoms of influenza infection, in the original manuscript, we also chose the histopathology of intestine as one index to evaluate the severity of influenza infection. However, in this experiment, the intestines of GX.DG were collected from moribund mice, so, as you said, the intestine histopathology might be due to the global circulation impairment in these severely infected mice. Regrettably, we neglected it in the original manuscript. So, we deleted the description about intestine histopathology in the revised manuscript, and corrected the Fig. 1 and Fig. S1.**

-The authors showed that fecal microbiota transfer using stool from mice at 3 or 9 days after infection had some effects on susceptibility to infection of recipient mice (microbiota of mice that will die from infection worsen the severity in recipients). Although this experiment is interesting, it is very difficult to analyze as the stools used for FMT come from mice already infected by influenza. It is thus likely that the worsening or protective effect is due to the reaction of the microbiota to the infection and not to a basal microbiota state. In the same way, the results of the control groups are difficult to interpret. It would be much easier to perform FMT with baseline (before infection) stool.

**Response: As you think, according to the procedure of our FMT, the worsening or protective effect indeed should be due to the reaction of the microbiota to the influenza infection, and not to a basal microbiota state. In fact, this is also our aim in this paper. We attempt to study whether the different changes of commensal microbiota caused by influenza infection can in turn influence the pathogenicity of influenza, and then screen the specific anti-influenza microbes. It is different from other studies about basal microbiota. So, in this study, FMT was performed via using the stools of infected mice. Additionally, on the issue about baseline control, your worry may be due to that our description is not very clear. Actually, we have already set one baseline control group, i.e., ‘NC donor’ group, in which FMT was performed using the stools of uninfected mice (NC mice). So, to avoid misunderstanding, we correct the related description. The amendments are highlighted in red in the revised manuscript.**

-All the results regarding the potential mechanism of action are speculative. Experimental validation would be needed to validate it.

**Response: As your suggestion, we verified the results of functional metagenomic analysis in mice. Considering CoA could be digested by gastric acid, we chose intraperitoneal injection as the administration method of CoA, while valine, isoleucine and lysine were administrated by gavage. The results indicated that oral administration of valine or intraperitoneal injection of CoA showed significant anti-influenza effect, by contrast, oral administration of lysine only improved the weight loss, and oral administration of isoleucine had no effect. At the same time, we also preliminary analyzed the mechanism through which valine and CoA mediate the anti-influenza effect. The data showed that both the treatments of valine and CoA can obviously reduce lung virus titers and alleviated the lung lesion, but can’t mediate the antiinfluenza effect of A549 cells in vitro, suggesting that the anti-influenza effects of valine and CoA might be indirect. Further, we found that, on day 5 post-infection, both oral administration of valine and intraperitoneal injection of CoA can significantly reduce the levels of IL-1β, IL-6, and IL-10 of lungs, and at the same time up-regulate IFN-β and IFN-γ of lungs. The difference is that, on day 0 post-infection, intraperitoneal injection of CoA can obviously induce the expressions of TNF-α, IL-1β, IL-6, and IFN-γ in lungs, but oral administration of valine only stimulates IFN-γ, suggesting that the antiinfluenza effect of valine could be due to its immunoregulatory properties after influenza infection, by contrast, that of CoA could be because it stimulates the innate immune response in advance. The supplementary data has been added in Fig. 6, and the related description has also been added in line “326-348” of the revised manuscript.**

-In figure 4, the mice are treated by antibiotics. So, the effect of the tested Bifidobacterium might not be specific. It would be more relevant to supplement non antibiotics treated mice with Bifidobacterium on a daily basis to evaluate the effect

**Response: As you said, some reports showed that pre-treatment with some antibiotics indeed does not facilitate establishment of exogenous microbiota, and then influence the specific functional evaluation. However, following constant attempts, this issue has been significantly improved by some broad-spectrum antibiotic regimens. Of which, our approach is “ampicillin (1 mg/mL), streptomycin (5 mg/mL), vancomycin (0.25 mg/mL), and colistin (1 mg/mL) were added to the sterile drinking water of mice for 3 days.” This approach has been applied in some other studies, and yielded very good results, for example, “Routy B, et al. Science. 2018. Gut microbiome influences efficacy of PD-1- based immunotherapy against epithelial tumors.” In addition, to eliminate any possible artifacts (e.g., residual microflora or the effect of antibiotics treatment), we also utilized germ-free mice to evaluate the effect of the tested Bifidobacterium for the influenza pathogenicity. Certainly, it is very meaningful to supplement non-antibiotics treated mice with Bifidobacterium on a daily basis to evaluate the effect. In fact, we also utilized non-antibiotics treated mice to evaluate the effect of the tested Bifidobacterium for the influenza pathogenicity, and obtained a similar result to that of antibiotics treated mouse model. The difference is that, compared with the former, the latter is more obvious. So, we only showed the results of antibiotics treated mouse model. Of course, if necessary, we can provide the results of non-antibiotics treated mouse model at any time.**

-There are many strains of B. animalis. Why this specific one has been chosen? It would have been more relevant to work with the one isolated from the animal facility.

**Response: You are right, it indeed has been more relevant to work with the one isolated from the animal facility. But in practice there are still many challenges and impediments. On the one hand, although Bifidobacterium is culturable, there are a great many of species and subspecies. So, screening specific B. animalis remains very difficult. On the other hand, isolated strains from different animal individuals probably exist differences, even though these animals come from one same community. So, one specific isolated strain can’t represent all isolated strains, whether it comes from the animal facility or other.**

**In addition, our data showed that B. animalis might mediate protective effect against influenza through promoting the biosynthesis of valine, isoleucine, lysine, and coenzyme A. Before animal experiment, our analysis found that the strain ATCC 25527 (B. animalis) also has the biosynthetic pathways of these molecules, providing some theoretical support for that we chose strain ATCC 25527 to validation.**

-DNA extraction method should be described more precisely. Was there a mechanical lysis step?

**Response: As your suggestion, we have added the more precise DNA extraction method in line “566-572” of revised manuscript.**

**We appreciate your warm work earnestly, and hope that the correction will meet with approval. Once again, thank you very much for your comments and suggestions.**

Reviewer #2: In the manuscript, entitled "Influenza infection elicits an expansion of gut population of endogenous Bifidobacterium animalis which protects the host against infection", authors identified gut commensal microbes that are responsible for antiinfluenza protection and verified their effects in defined animal models. Experimental procedures are robustly provided and microbial population analysis was well performed. Authors also proposed a potential mechanism of protection by functional metagenomic analysis. Overall, results are of interest in that changes in commensal microbial population induced their host to be more resistant to viral infection. However, presented results are still at the preliminary stage, and further additional results should be provided to corroborate authors' claims.

**Response: Special thanks to you for your good comments. As your suggestion, we supplemented a series of experiments, including qRT-PCR of B. pseudolongum- and B. animalis- specific genes, evaluation of anti-influenza effect of valine, isoleucine, lysine and CoA, and so on. It's worth mentioning that, we demonstrated the antiinfluenza effects of valine and CoA, and also preliminary analyzed their anti-influenza mechanism. These data supported the results of functional metagenomic analysis, and further revealed the anti-influenza mechanism of B. animalis, providing strong evidence for our claims. All amendments are highlighted in red in the revised manuscript. At the same time, the responses for your comments have also been listed below.**

1.Based on Fig. 1A, GX-infected mice begun to die at 9 days post-infection. How could authors collect feces from GX.DG donor at 3 days post-infection as illustrated in Fig. 1E?

**Response: As shown in Fig. 1E, the feces were collected from GX-infected mice at day 3 and 9 post-infection respectively, at that time, we did not divide the feces from each mouse into GX.SG or GX.DG. In order to the subsequent grouping, firstly, within 2 h of collection, the fecal microbes from each mouse were resuspended in PBS with 10% (v/v) glycerol and frozen at −80°C respectively. After 15 days infection, combined with the ear-mark, then the pre-collected fecal microbe samples were divided into two groups based on whether the mice died or survived the infection. The more precise description has been added in line “500” of the revised manuscript.**

2.Authors performed co-abundance genes (CAGs) analysis to provide microbial population at the species level. Since Bifidobacterium animalis is culturable, it needs to be shown that Bifidobacterium animalis population is actually enriched in GX.SG vs. GX.DG. Authors can try to cultivate fecal matters discharged from GX.SG and GX.DG groups and measure changes in B. animalis CFUs on a Bifidobacterium selective agar plates. Alternatively, quantitative RT-PCR can be performed to compare the copynumber of B. animalis-specific genes.

**Response: Thank you for your important comments. As your suggestion, we performed the qRT-PCR to compare the copy-numbers of B. pseudolongum- and B. animalisspecific genes respectively. As a result, we obtained consistent conclusion with CAGs analysis, i.e., both B. pseudolongum and B. animalis were enriched in GX.SG vs. in GX.DG at day 2 and 10 post-infection, but not at day 0 post-infection. The supplementary data has been added in Fig. S8, and the related description has also been added in line “234-238” of the revised manuscript.**

3.Influenza virus is infected via intranasal route. FMT affects microbial population changes in the gut. Additional experiments are necessary to evaluate events that occurred through this gut-airway axis. Images shown in Fig. 4F do NOT clearly show that improved lung pathology in mice administered with B. animalis alone or with both.

**Response: You are right, the events that occurred through this gut-airway axis are very complex. More experiments contribute to more clearly understand the gut-airway axis. However, the studies about gut-airway axis are still limited up to now. Especially, which test indexes are required remains unclear, and at the same time, applicable test indexes are different for different experimental models. In a previous study about gut microbiota and influenza infection, “Stephan P. Rosshart, et al. Cell. 2017. Wild mouse gut microbiota promotes host fitness and improves disease resistance”, authors mainly evaluated survival rate, body weight change, lung viral titer, cytokines, and lung histology. As a reference, in this study, we also evaluated similar indexes.**

**In addition, about the issues of Fig. 4F, it is possibly because the lung pathology of positive control mice at day 5 post-infection is not obvious enough. In order to solve this problem, we performed new animal experiment and altered the sampling time of lung. In the new animal experiment, the lungs were re-collected at day 0 and 7 postinfection for the histological examination, respectively. The results showed that the lungs of mice administered with PBS or B. pseudolongum alone displayed severe pathologies, including congestion, inflammatory cells infiltration, and deciduous cells in the bronchial lumen, even alveoli almost disappeared. By contrast, the lung lesions of mice administered with B. animalis alone or the two-bacterium combination were obviously improved. The revised images can clearly show that improved lung lesion in mice administered with B. animalis alone or with both. At the same time, the corresponding amendments have been highlighted in red in the revised manuscript.**

4.Authors claimed that B. animalis-mediated anti-viral effects are due to the biosynthesis of valine, isoleucine, lysine and coenzyme A. But, no supporting evidence is provided. To be able to claim that argument, authors need to perform additional experiments. For example, these molecules are chemically provided into the mouse airway and test the effects. In addition, authors need to provide reasonable explanation how these molecules can be delivered to the airway or if not, how these molecules strengthen the anti-viral responses.

**Response: As your suggestion, we verified whether valine, isoleucine, lysine and CoA have anti-influenza effect. Considering CoA could be digested by gastric acid, we chose intraperitoneal injection as the administration method of CoA, while valine, isoleucine and lysine were administrated by gavage. The results indicated that oral administration of valine or intraperitoneal injection of CoA showed significant antiinfluenza effect, by contrast, oral administration of lysine only improved the weight loss, and oral administration of isoleucine had no effect. At the same time, we also preliminary analyzed the mechanism through which valine and CoA mediate the antiinfluenza effect. The data showed that both the treatments of valine and CoA can obviously reduce lung virus titers and alleviated the lung lesion, but can’t mediate the anti-influenza effect of A549 cells in vitro, suggesting that the anti-influenza effects of valine and CoA might be indirect. In fact, we also evaluate the anti-influenza effects of valine, isoleucine, lysine and CoA in mice by nasal drip, unfortunately, we didn’t find any anti-influenza effect. This further supported our above speculation that the antiinfluenza effects of valine and CoA might be indirect. Further, we found that, on day 5 post-infection, both oral administration of valine and intraperitoneal injection of CoA can significantly reduce the levels of IL-1β, IL-6, and IL-10 of lungs, and at the same time up-regulate IFN-β and IFN-γ of lungs. The difference is that, on day 0 postinfection, intraperitoneal injection of CoA can obviously induce the expressions of TNFα, IL-1β, IL-6, and IFN-γ in lungs, but oral administration of valine only stimulates IFN γ, suggesting that the anti-influenza effect of valine could be due to its immunoregulatory properties after influenza infection, by contrast, that of CoA could be because it stimulates the innate immune response in advance. The supplementary data has been added in Fig. 6, and the related description has also been added in line “326-348” of the revised manuscript.**

**We appreciate your warm work earnestly, and hope that the correction will meet with approval. Once again, thank you very much for your comments and suggestions.**

**Second round of review**

**Reviewer 1**

The authors adressed my comments.
I am only concerned by the DNA extraction method that does not include a mechanical lysis step.

**Reviewer 2**

Comments are addressed to my satisfaction and the revised manuscript has been improved.
